# Molecular Determinants of EphA2 and EphB2 Antagonism Enable the Design of Ligands with Improved Selectivity

**DOI:** 10.1021/acs.jcim.3c01064

**Published:** 2023-11-01

**Authors:** Lorenzo Guidetti, Alfonso Zappia, Laura Scalvini, Francesca Romana Ferrari, Carmine Giorgio, Riccardo Castelli, Francesca Galvani, Federica Vacondio, Silvia Rivara, Marco Mor, Chiara Urbinati, Marco Rusnati, Massimiliano Tognolini, Alessio Lodola

**Affiliations:** † Dipartimento di Scienze degli Alimenti e del Farmaco, Università degli Studi di Parma, Parco Area delle Scienze 27/A, I- 43124 Parma, Italy; ‡ Microbiome Research Hub, Università degli Studi di Parma, Parco Area delle scienze 11/A, I- 43124 Parma, Italy; § Dipartimento di Medicina Molecolare Traslazionale, Università degli Studi di Brescia, Brescia 25121, Italy

## Abstract

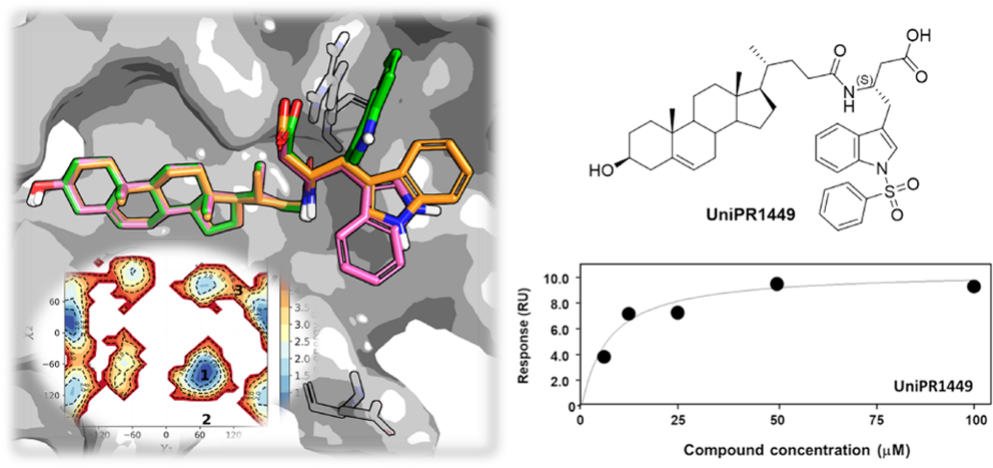

With the aim of identifying novel antagonists selective for the EphA receptor family, a combined experimental and computational approach was taken to investigate the molecular basis of the recognition between a prototypical Eph–ephrin antagonist (UniPR1447) and two representative receptors of the EphA and EphB subfamilies, namely, EphA2 and EphB2 receptors. The conformational free-energy surface (FES) of the binding state of UniPR1447 within the ligand binding domain of EphA2 and EphB2, reconstructed from molecular dynamics (MD) simulations performed on the microsecond time scale, was exploited to drive the design and synthesis of a novel antagonist selective for EphA2 over the EphB2 receptor. The availability of compounds with this pharmacological profile will help discriminate the importance of these two receptors in the insurgence and progression of cancer.

## Introduction

The erythropoietin-producing hepatocellular carcinoma (Eph) receptors represent the largest family of tyrosine kinases and comprise 14 subtypes divided in two classes, EphA and EphB, according to sequence homology and their affinity for membrane-anchored proteins, known as ephrins.^1^ EphA receptors (EphA1-A8, EphA10) are recognized and activated by ephrin-A ligands (ephrin-A1-A5), while EphB receptors (EphB1-EphB4, EphB6) are engaged by three ephrin-B ligands (ephrin–B1-B3). A distinctive feature of the Eph–ephrin system is the ability to produce bidirectional signals after an Eph–ephrin interaction has occurred at the cell–cell interface.^2^

**Figure 1:**
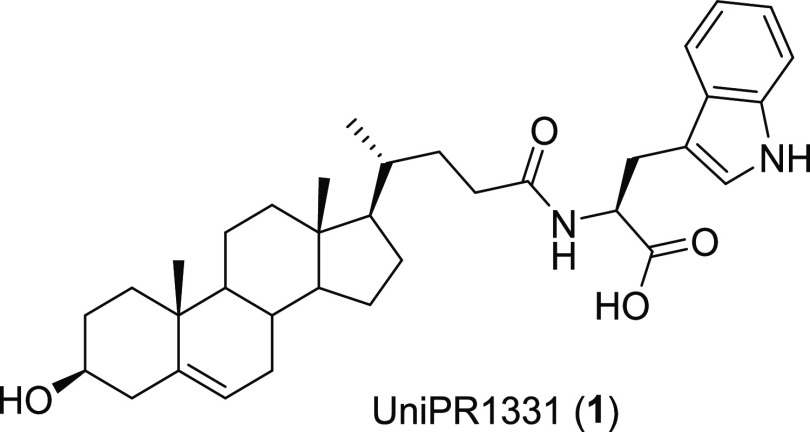
Chemical structure of the Eph–ephrin antagonist UniPR1331.

The formation of the Eph–ephrin heterodimeric complex is a key signaling event in several physiological processes,^3^ including embryogenesis and adult tissue regeneration.^4^ On the other hand, the deregulation of the Eph–ephrin signaling system in the adult, including alteration of EphA2^5^ and EphB2^6^ expression, has been correlated with the insurgence and progression of cancer disease.^7,8^ The EphA2 receptor has attracted particular attention in a drug discovery perspective,^9,10^ as its activity has been linked to tumoral angiogenesis and vasculo-mimicry,^11^ insurgence of metastasis,^12^ and the maintenance of the cancer stem niche.^13^ Additionally, the inhibition of the EphA2–ephrin-A1 signaling axis^14,15^ has been shown to reduce growth of different solid tumors,^16^ including glioblastoma multiforme (GBM),^17^ a rare central nervous system (CNS) disease for which effective treatments based on a molecularly targeted approach are still missing.^18^ In this scenario, different strategies have been applied to the search for small molecules able to disrupt the Eph–ephrin complex^19,20^ leading to the discovery of the l-tryptophane conjugate of 3β-hydroxy Δ^5^-cholenic acid^21^ (UniPR1331,^22^Figure 1). This is the only reported brain penetrant small molecule inhibiting the ectodomain of EphA2 that has been shown to reduce tumor growth in *in vivo* models of GBM, with a remarkable delay in the time to progression of the disease.^23^ Other experiments have also shown that this compound not only inhibits EphA2 but also interferes with the activity of several other members of the Eph receptor family, including the EphB2 receptor subtype, known to be an oncogenic driver of GBM.^24,25^ In the present scenario, the availability of a second generation of 3β-hydroxy Δ^5^-cholenic acid conjugates with improved selectivity might promote our understanding of the importance of different Eph-dependent signals in GBM.^26^

**Figure 2:**
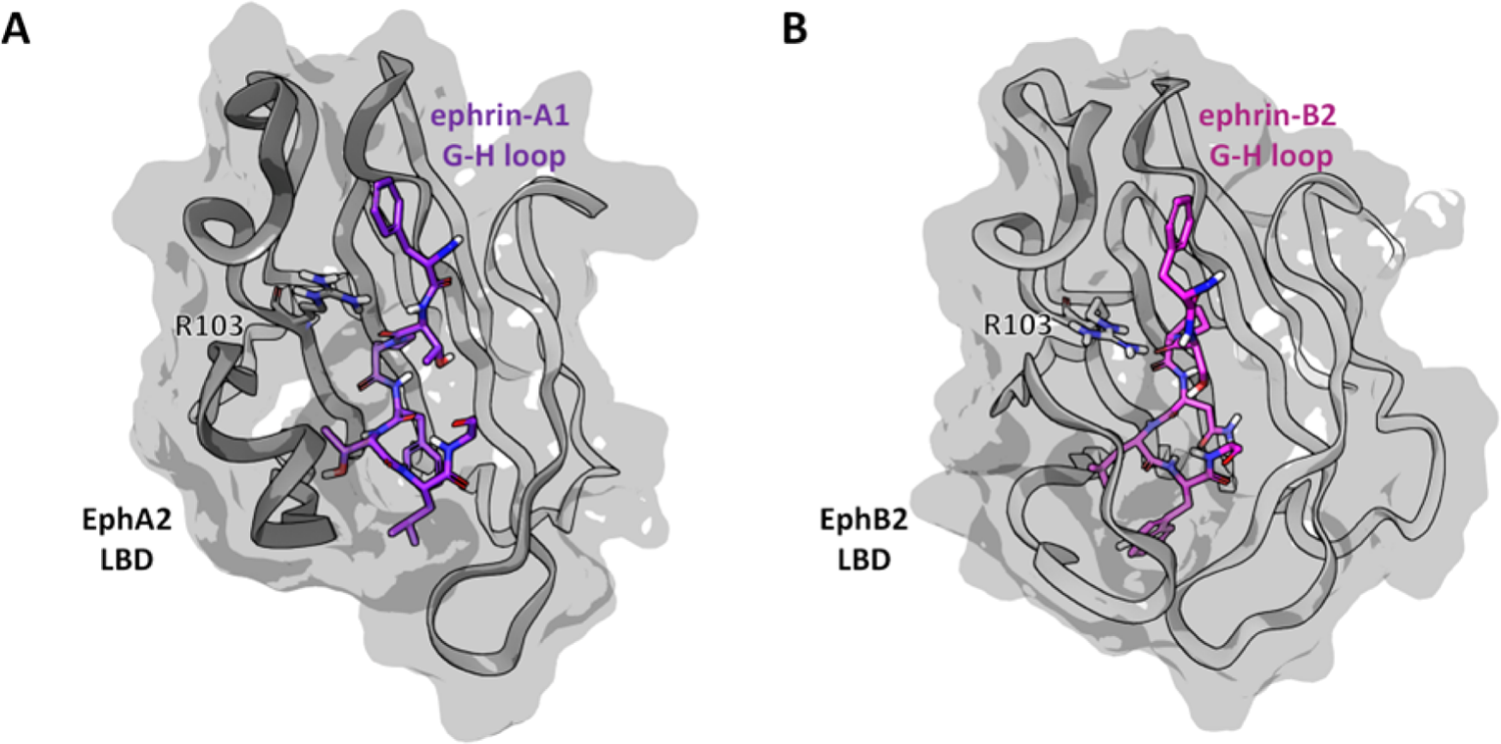
Visualization of the ligand binding domain of EphA2 (panel A, gray carbon atoms and ribbons) and EphB2 (panel B, gray carbon atoms and white ribbons) complexed within ephrin-A1 (left, violet carbon atoms) and ephrin-B2 (pink carbon atoms) as observed in the X-ray structures reported by Himanen and colleagues.^27^ The conserved residue Arg103 is also displayed in both receptor subtypes.

Visual inspection of the X-ray coordinates of EphA2^27^ reveals that its ligand binding domain (LBD)^28^ possesses a β-sandwich jellyroll fold composed of 11 antiparallel β-strands connected by a set of loops of different lengths. Both β-strands and loops contribute to the definition of the Eph ligand binding cleft. The LBD of EphB2 possesses a similar fold^29^ (EphB2 shares 30% sequence identity with EphA2), and it can be easily superimposed on the EphA2 LBD, with a root-mean-square deviation (RMSD) lower than 2.0 Å. Analysis of the two binding sites reveals the presence of similar features including (i) a hydrophobic cavity that recognizes a conserved sequence enriched in lipophilic amino acids (i.e., FTPFTLG in ephrin-A1; FSPNLWG in ephrin-B2; see Figure 2) present in the G-H loop of ephrin ligands; and (ii) the so-called *latch* residue (Arg103 in both EphA2 and EphB2 receptor subtypes), which contributes to the recruitment of the ephrin ligands through the formation of a salt bridge with a conserved acid residue (not displayed in Figure 2).

Extensive structure–activity relationship (SAR) data and docking simulations suggest that UniPR1331 binds EphA2 accommodating its steroidal core within a wide hydrophobic channel on the surface of the EphA2 receptor, with its carboxylic group forming a key salt bridge with the highly conserved residue Arg103.^22^ A similar binding mode can be proposed also for the interaction involving UniPR1331 and EphB2, somehow indicating that Eph receptor subtype selectivity could be hardly achieved in the series of 3β-hydroxy Δ^5^-cholenic acid amino acid conjugates. Overall, the similarity between EphA2 and EphB2 binding sites and between ligand binding modes has prevented the discovery of selective agents.

In the present work, we started our investigation by synthesizing and testing the Eph–ephrin system, UniPR1447, a close analogue to UniPR1331, in which the L-tryptophan has been replaced by the L-β-homotryptophan. Biochemical assays and simulations were applied to obtain hints useful for achieving selectivity. Crucially, simulations pointed to the presence of an accessory binding pocket in EphA2 that was exploited to synthesize a new antagonist, resulting in selectiveness for the EphA2 receptor over the EphB2 one. Our results confirm that a comprehensive exploration of the conformational space of a ligand in complex with its target can give crucial information for the optimization of ligands targeting the Eph–ephrin system.^30,31^

## Results and Discussion

### Molecular Characterization of UniPR1447 as a Dual EphA2 and EphB2 Antagonist

**Scheme 1:**
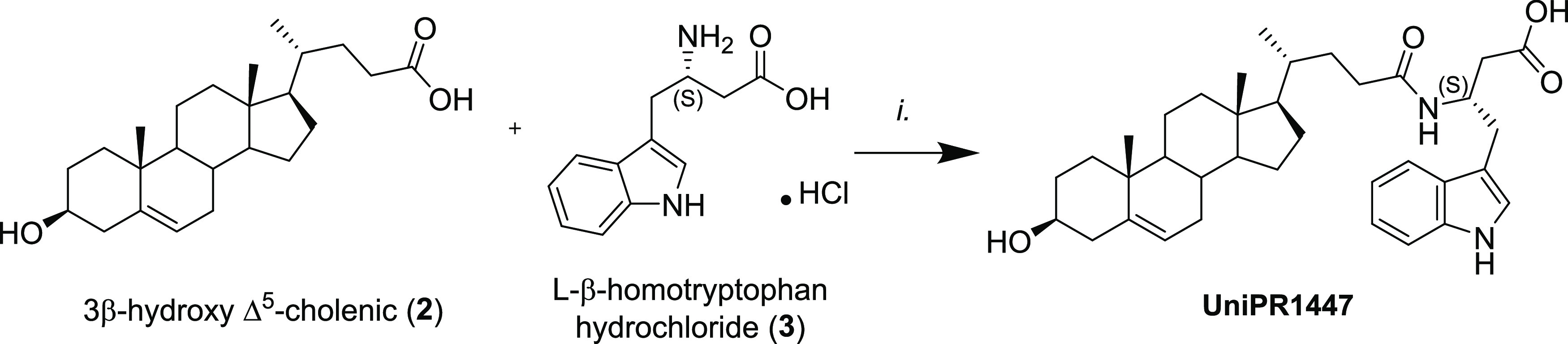
Reagents and Conditions: (i) DIPEA 5 equiv, TBTU 1.2 equiv, Anhydrous. DMF, 0 °C, 30 min

We started our study synthesizing the L-β-homotryptophan conjugate of 3β-hydroxy Δ^5^-cholenic acid (UniPR1447). In brief, by exposure of a chilled solution of 3β-hydroxy Δ^5^-cholenic in DMF to 1.2 equiv of *O*-(benzotriazol-1-yl)-*N*,*N*,*N*′,*N*′-tertamethyluronium tetrafluoroborate (TBTU), the activated *O*-benzotriazol-1-yl-ester was formed. Without isolation, direct addition of L-β-homotryptophan hydrochloride produced the conjugates UniPR1447 in good yield (65%), after silica gel column chromatography (Scheme 1).

**Figure 3:**
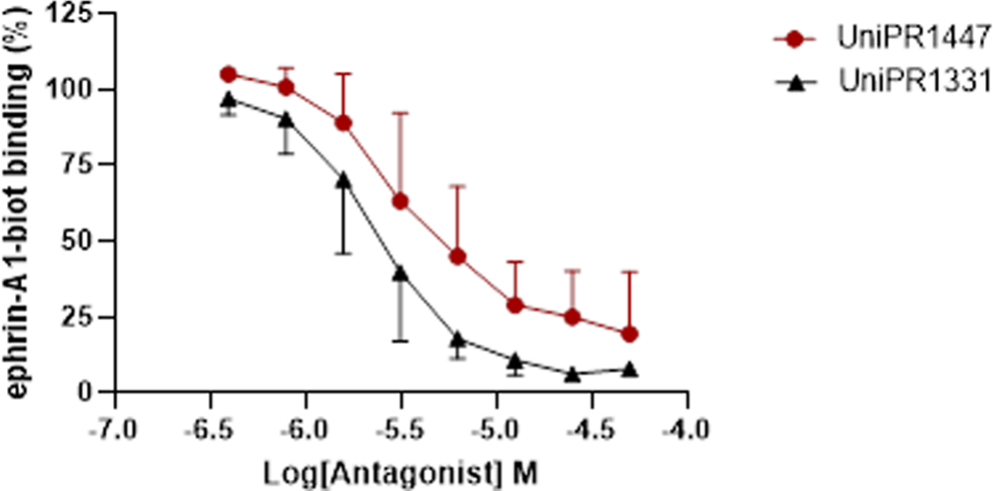
Concentration–response curves for UniPR1447 and UniPR1331 in the displacement assay of biotinylated ephrin-A1-Fc from EphA2-Fc. Compounds were added to the wells at proper concentrations in 0.5% DMSO and incubated at 37 °C for 1 h. Biotinylated ephrin-A1-Fc was added at 37 °C for 4 h. Data are the means of three independent experiments ± standard deviation.

We next evaluated whether the new compound could inhibit the binding of biotinylated ephrin-A1-Fc to the EphA2 receptor through an ELISA-binding assay. Displacement curves were constructed using increasing concentrations of the ligand (from 1.6 to 50 μM) in the presence of biotinylated ephrin-A1-Fc at a concentration corresponding to its *K*_D_ value. UniPR1447 was able to significantly prevent EphA2–ephrin-A1 binding in a concentration-dependent manner (Figure 3). The resulting value of the half-maximal inhibitory concentration (IC_50_) was 6.6 μM, in line with that of the reference antagonist UniPR1331 (IC_50_ = 2.6 μM).

**Figure 4:**
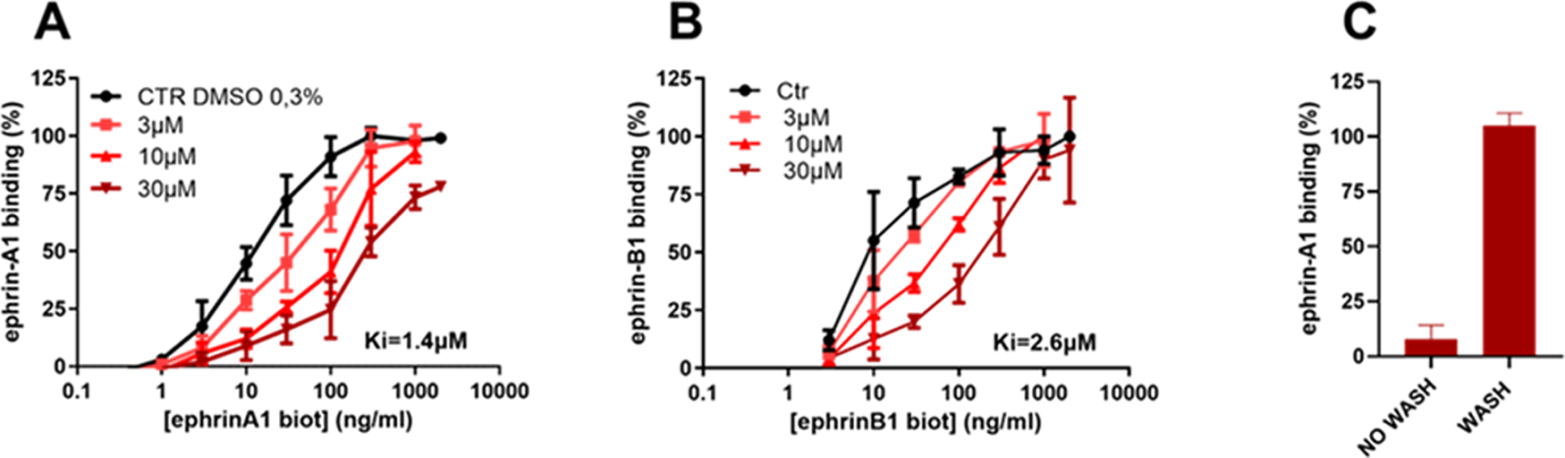
Binding of biotinylated ephrin-A1-Fc to immobilized EphA2 (A) and of biotinylated ephrin-B1-Fc to immobilized EphB2 (B) in the presence of different concentrations of UniPR1447. EphA2–ephrin-A1 binding in the presence of 50 μM UniPR1447 with or without washing three times with PBS before the addition of biotinylated ephrin-A1-Fc (C). Data are the means of at least three independent experiments ± the standard deviation.

We next evaluated the ability of UniPR1447 to prevent biotinylated ephrin-B1-Fc from binding to EphB2, observing a similar potency (Figure S1, SI). Considering the ability of UniPR1447 to interfere with ephrin binding to both EphA2 and EphB2 receptors, we built saturation curves of biotinylated ephrin-A1-Fc on the EphA2 receptor and biotinylated ephrin-B1-Fc on the EphB2 receptor in the presence of increasing concentrations (from 3 to 30 μM) of UniPR1447 (Figure 4). We retrieved inhibitory constant (*K_i_*) values for both receptors by means of a nonlinear regression analysis, which allowed us to have a more robust estimation of the affinity of this compound for receptors under consideration than simple IC_50_ values. UniPR1447 displayed a similar affinity for both receptors, with *K_i_* values of 1.4 μM for EphA2 and 2.6 μM for EphB2, respectively. The binding of the compound to EphA2 (Figure 4C) and EphB2 (data not shown) was fully reversible. This investigation showed the ability of L-β-homoamino acid conjugates of 3β-hydroxy Δ^5^-cholenic acid to inhibit both the EphA2 and EphB2 receptors with similar potency.

### Site Map Analysis of EphA2/EphB2 and Investigation of the Binding Modes of UniPR1447

**Figure 5:**
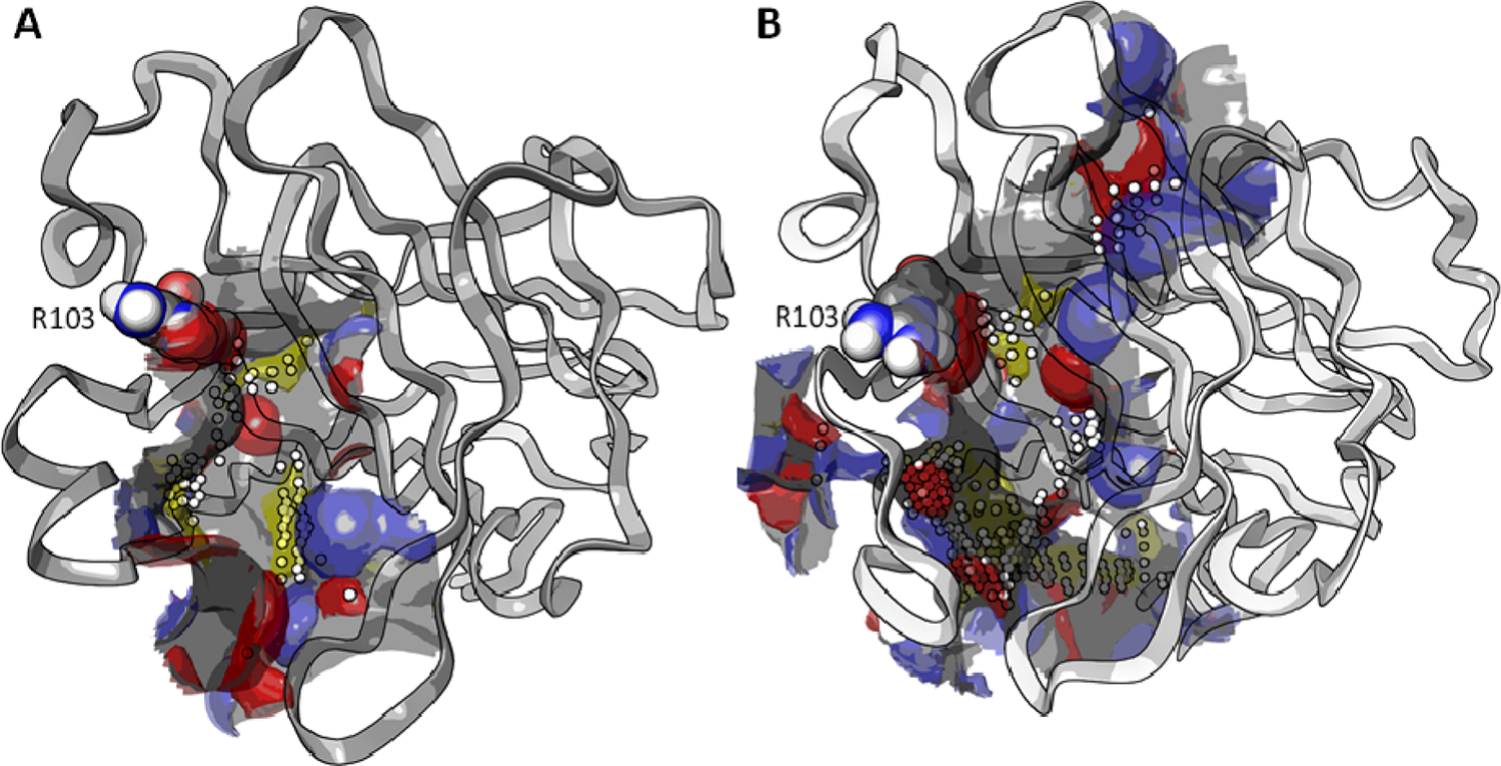
Analysis of EphA2 (panel A, gray ribbons) and EphB2 (B, white ribbons) LBD. White spheres represent shallow solvent exposed regions in the receptor, while the gray surface represents the overall binding site, including buried regions. Hydrophobic (yellow), H-bond donor (blue), and H-bond acceptor (red) maps are mapped on the receptor surface. Arg103 is represented with CPK model.

We continued our investigation by analyzing the features of the high-affinity binding sites present in LBD of EphA2 and EphB2 using *SiteMap*.^32^ This computational tool is able to identify binding sites by linking together “site points” that are most likely to contribute to protein–ligand association on the basis of mutual proximity and shielded from solvent.^33^ Analysis of the binding site showed that the volume of the EphB2 binding site (570 Å^3^) was larger than that of EphA2 (300 Å^3^) with the presence of an accessory pocket above the conserved residue Arg103 (Figure 5). On the other hand, visual inspection of the structures showed that the solvent exposed region juxtaposed to Arg103 was more open in the case of EphA2 than for EphB2 due to a different arrangement of the β-strand C, which contains the WDLMQNI sequence in the former and the WEEVSGY sequence in the latter (Figure S2, SI). The replacement of an aspartate (Asp53, EphA2) with a glutamate (Glu52, EphB2) in this β-strand reduced the available space in this region of the LBD of EphB2. These differences are somehow unexpected, considering that the G-H loop of the ephrin-A1 and ephrin-B2 ligands, both protruding within the LBD of EphA2 and EphB2, possesses similar steric hindrance and molecular properties. It could be speculated that the β-strand C region could be exploited to drive selectivity versus one of the two isoforms.

**Figure 6:**
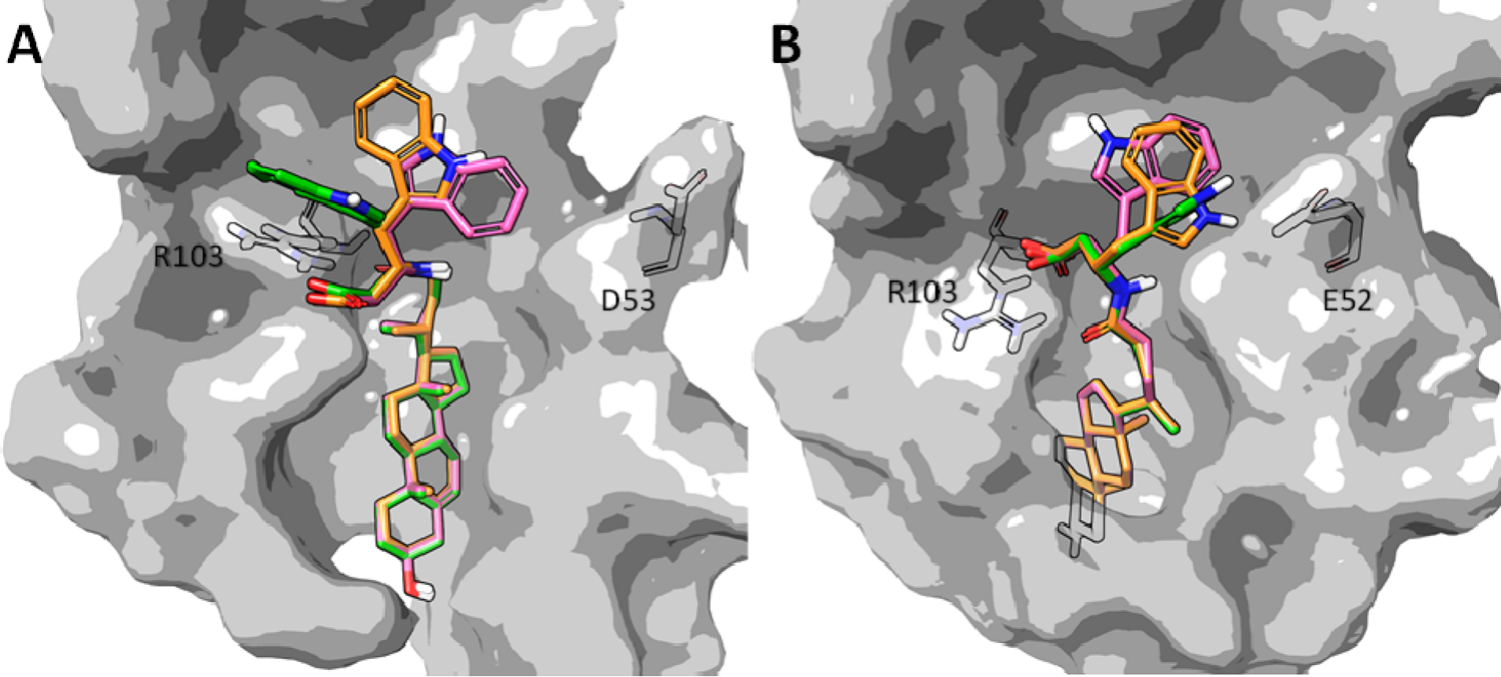
Top-ranked binding poses of UniPR1447 (orange carbon atoms, 1st ranked; green carbon atoms, 2nd ranked; pink carbon atoms, 3rd ranked) within the LBD of the EphA2 (left panel, gray surface) and EphB2 receptors (right panel, light-gray surface).

Considering the competitive antagonism displayed by UniPR1447 in the biochemical assay reported above, we evaluated possible binding modes assumed by it within the LBD of EphA2 and EphB2 through docking simulations using Glide.^34,35^ Analysis of the best-ranked poses showed that UniPR1447 adopted a comparable binding mode within EphA2 and EphB2, with the 3β-hydroxy Δ^5^-cholenic acid scaffold placed within the wide hydrophobic channel usually targeted by the G-H loop of ephrin ligands, and the carboxylate group of the L-β-homotryptophan moiety pointing toward the side chain of a highly conserved arginine residue (Arg103 in both EphA2 and EphB2, Figure 6). The importance of this salt bridge has been previously tested through the synthesis of the methyl ester analogue of UniPR1331, which resulted inactive on the Eph–ephrin system when tested up to 100 μM.^22,36^

Alternative accommodations were identified for the indole ring of UniPR1447 due to the flexibility of the side chain of the amino acid portion. In the identified poses, χ1 and χ2 dihedral angles assumed different values without significantly affecting the docking score (Table 1). In both of the receptors, the best solution found was characterized by an arrangement of the indole ring in which its polar -NH group pointed toward the β-strand C of the LBD, which contained a lipophilic stretch except from Asp53 in EphA2 and Glu52 in EphB2.

**Table 1:**
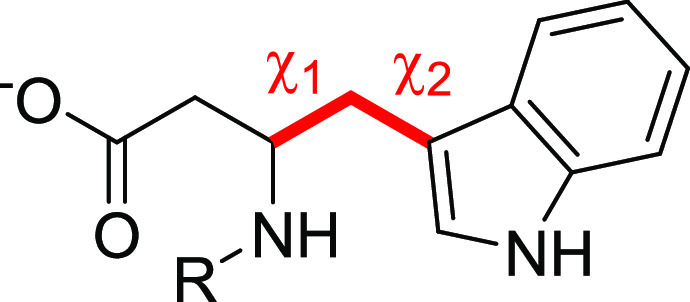
Top-Ranked Binding Poses within EphA2 and EphB2 Receptors for UniPR1447

ranking	receptor	χ_1_ (degrees)	χ_2_ (degrees)	score (kcal/mol)
1	EphA2	–57	–15	–5.5
2	EphA2	–171	58	–5.0
3	EphA2	–62	155	–4.8
1	EphB2	60	–91	–7.4
2	EphB2	63	–176	–6.5
3	EphB2	120	72	–6.3

### Molecular Simulations of the UniPR1447 in Complex with EphA2 and EphB2 Receptors

Even though docking can generate plausible binding modes at a minimal computational cost,^37,38^ this approach cannot provide an exhaustive exploration of the conformational space of a ligand in complex with its target.^39^ If performed in the microsecond time scale,^40,41^ unbiased MD simulations allow one to characterize the binding basin of a ligand within the target binding site.^42,43^ In addition, if alternative binding modes are separated by low-energy barriers, unbiased MD simulations can easily access these configurations.^44^ By experiencing an adequate number of transitions between alternative binding modes, the relative abundance of each state obtained by frequency analysis can be used to reconstruct the conformational free-energy of binding, similarly to what was performed in ref (45).

To sample the conformational space of UniPR1447 within the binding pocket of EphA2 and EphB2, specifically focusing on the accessible conformations of the L-β–homotryptophan portion, we thus performed MD simulations on the docking poses of the ligand on both receptor subtypes. Two variables, χ1 and χ2, were selected to monitor the rotation of the rotatable bonds describing the position of the indole ring within the LBD of both EphA2 and EphB2 receptors. Similar approaches have been applied to explore the conformational space of biologically active ligands in the condensed phase to rationalize protein–ligand binding^46^ or to obtain key insights for drug design.^47,48^

**Figure 7:**
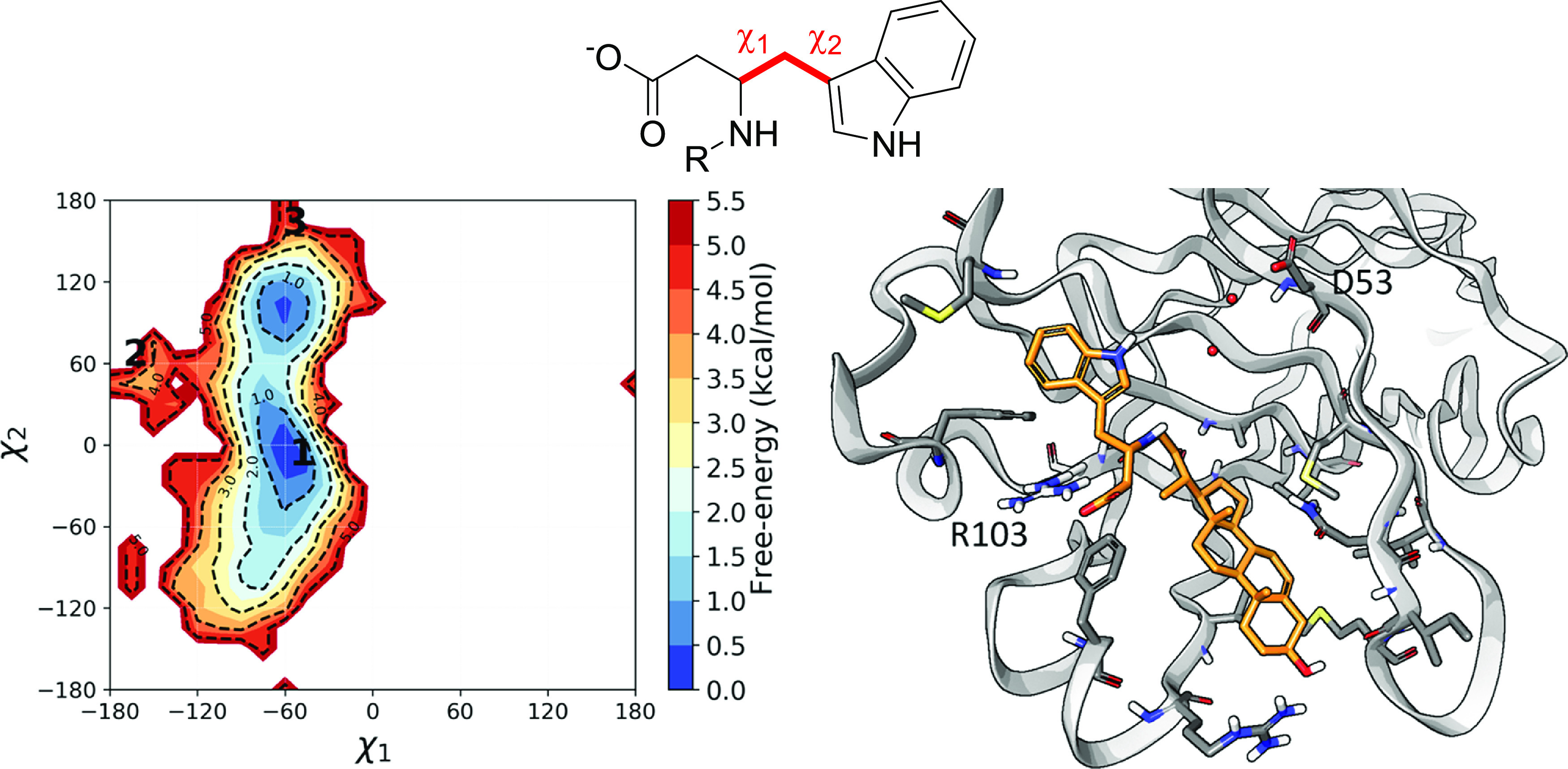
Free-energy conformational surface reconstructed along χ1 (N–Cα-Cβ-Cγ) and χ2 (Cα-Cβ-Cγ-Cδ1) after a 2.5 μs long MD simulation for the EphA2–UniPR1447 complex (left panel) and representation of the global minima binding conformation (right panel, EphA2 gray carbon atoms; UniPR1447, orange carbon atoms) with the indole -NH group pointing toward a solvent exposed cavity (crystallographic waters in red) delimited by Asp53. 1, 2, 3 labels on the FES represent the positions of docking poses projected on χ1 and χ2 spaces.

Analysis of a 2.5 μs MD simulation of EphA2 in complex with UniPR1447 showed that the ligand experienced transitions between alternative configurations, as indicated by the temporal evolution of χ1 and χ2 dihedral angles (Figure S3, SI). The global minimum corresponded to the best-ranked configuration found by docking (pose 1), with χ1 and χ2 values close to −60 and 0°, respectively. In this arrangement, the -NH group of the indole ring was projected toward a side pocket present in the EphA2 receptor delimited by Asp53, belonging to β-strand C, and filled by water molecules (Figure 7), as also observed in several X-ray structures of the EphA2 receptor ligand binding domain. It is worth noting that docking poses 2 and 3 resulted as high-energy states according to conformational free-energy surface (FES) reconstructed from the MD simulation. The same FES indicated the presence of an alternative minimum, featuring a free-energy content higher than that of the global minimum by nearly 1.0 kcal/mol. This alternative basin was characterized by the same χ1 value of the global minimum but of a χ2 value close to 90°. In this arrangement, the indole -NH group pointed toward the surface of the protein LBD delimited by a lipophilic region delimited by the disulfide bridge formed by Cys70 and Cys188 (Figure S4, SI).

**Figure 8:**
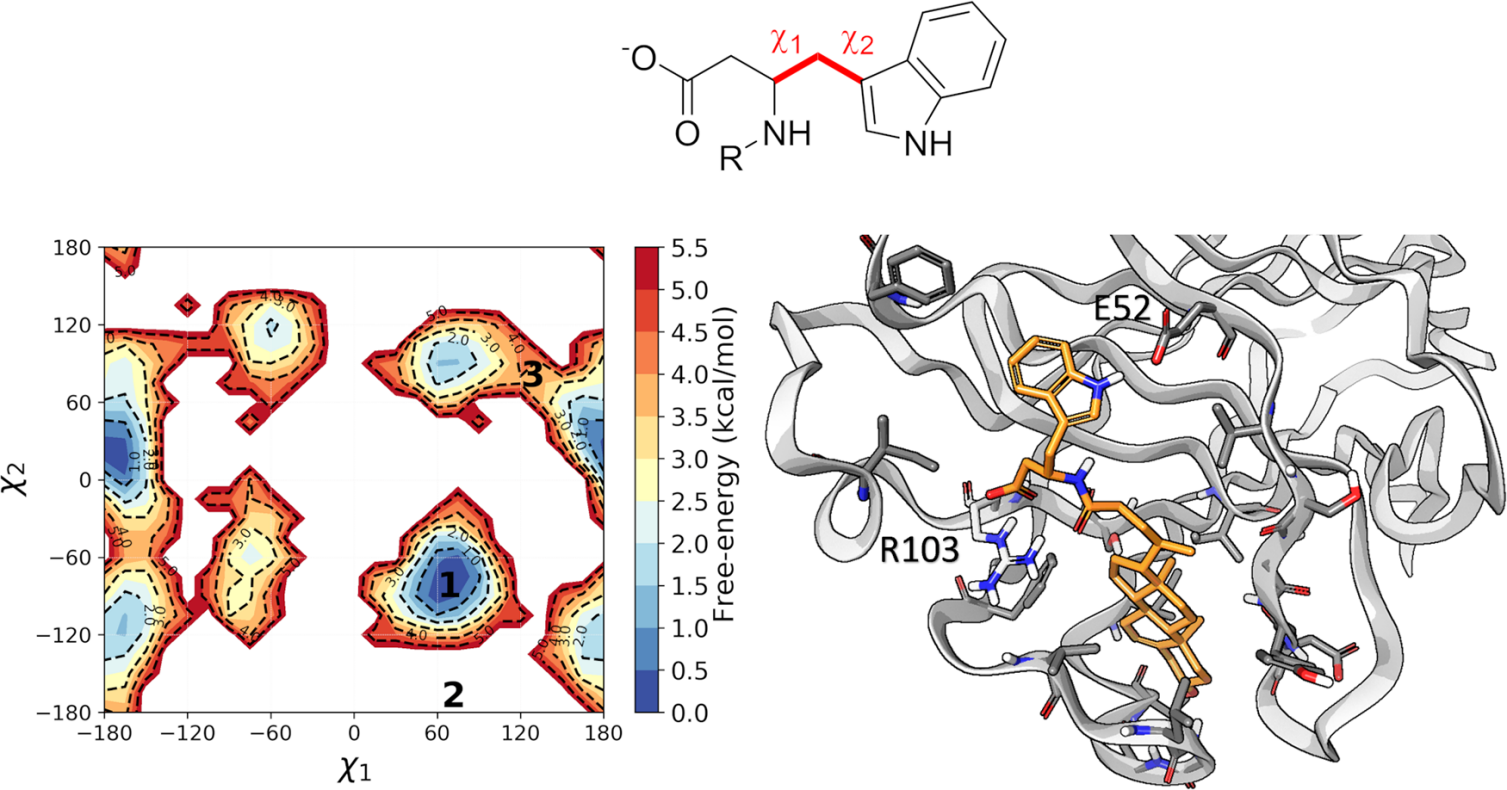
Free-energy conformational surface reconstructed along χ1 (N–Cα-Cβ-Cγ) and χ2 (Cα-Cβ-Cγ-Cδ1) after 2.5 μs long MD simulations for the EphB2–UniPR1447 complex (left panel) and representation of the global minimum binding conformation (right panel, EphB2 gray carbon atoms; UniPR1447, orange carbon atoms) with the indole -NH group forming a H-bond with Glu52. 1, 2, 3 labels on the FES represent the positions of docking poses reported in Figure 5 projected on χ1 and χ2 space.

The convergence of the plain MD simulation was assessed by evaluating the evolution of the FES versus the simulation time. The simulation appeared to converge after 1 μs, as no significant variations occurred in both position and relative energies of minima (Figure S5). Two other 2.5 μs long replicas were performed, leading to comparable FESs in qualitative terms (i.e., within the 1.5 kcal/mol uncertainty limit), indicating that binding pose 1 corresponded to a well-defined free-energy minima (Figure S6). Also, in the case of EphB2 in complex with UniPR1447, the ligand visited alternative binding arrangements at the level of the homotryptophan portion during MD simulation (Figure S7). The analysis of the FES reconstructed by frequency analysis from the MD trajectory showed that the global minimum corresponded to the best-ranked configuration found by docking (Figure 6). On the contrary, the other docking poses found by Glide resulted in high-energy configurations on the FES, separated by the global minimum by 5 or more kcal/mol. Other minima were identified, but these were less populated than the configuration corresponding to the top-ranked docking pose. Such a preference could be attributed to the presence of a H-bond occurring between the NH of the indole ring and the side chain of Glu52 emerging from the β-strand C of EphB2 (Figure 8). Also in this case, two other 2.5 μs MD replicas were performed, and the convergence of simulations was assessed by evaluating the evolution of the FESs throughout the simulation time. The resulting FESs indicated that binding pose 1 corresponded to a well-defined free-energy minimum (Figures S8 and S9).

### Design, Synthesis, and Characterization of a New EphA2 Antagonist

The preferred binding conformation identified by μs-long MD simulations corresponded to an arrangement of the L-β-homotryptophan moiety that would allow the installation of a bulky substituent at the indole nitrogen atom in EphA2 (Figure 7, right panel) but not in EphB2 (Figure 8, right panel). Docking simulations supported this hypothesis (*vide infra*), as aromatic substituents placed at nearly 90° with respect to the indole ring could be easily accommodated into an accessory pocket delimited by Asp53 in EphA2. The absence of such a cavity in EphB2 might prevent the generation of productive docking poses, i.e., comparable to the binding mode reported for the parent compounds UniPR1447, indicating that the replacement of indole -NH hydrogen with bulky substituents should be detrimental for activity on EphB2.

**Scheme 2:**
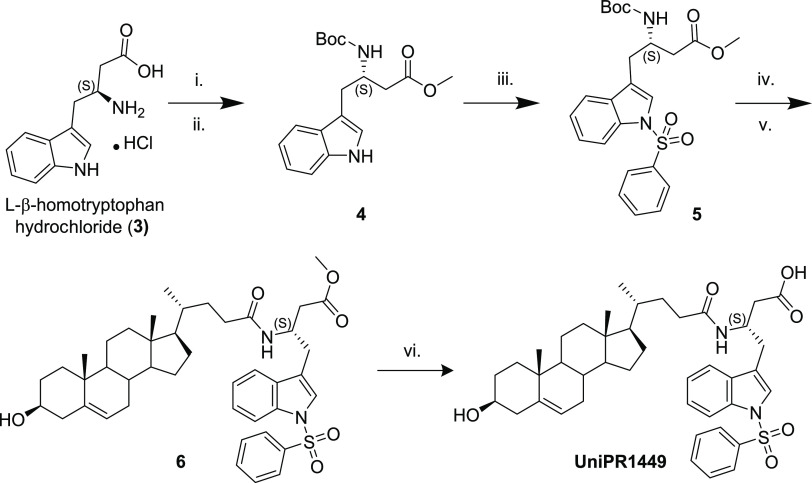
Reagents and Conditions: (i) MeOH, SOCl_2_; (ii) Boc_2_O, NaHCO_3,_ THF/H_2_O; (iii) PheSO_2_Cl, NaOH, N(Bu)_4_Cl, DCM/H_2_O; (iv) TFA/DCM, 0 °C; (v) 3β-hydroxy-Δ^5^cholenic acid, TBTU, DIPEA, DMF; (vi) LiHMDS, THF/H_2_O

To synthesize the *N*-sulfonylphenyl derivative of UniPR1447, as depicted in Scheme 2, we devised a few steps of protecting group manipulation on the commercially available L-β-homotryptophan hydrochloride. The carboxylate group was converted to a methyl ester in quantitative yield, and the crude product was converted to the *tert*-butyl carbamate (Boc-) on the amine function in 80% yield. Sulfonylation of the obtained protected L-β-homotryptophan derivative was performed under phase transfer conditions (DCM/H_2_O, Bu_4_NCl, NaOH, 46% yield). The obtained *N*-indole-sulfonylated product was then deprotected from the Boc- protecting group by treating with chilled trifluoroacetic acid and, without purification, subjected to condensation with 3β-hydroxy Δ^5^-cholenic acid with 2-(1*H*-benzotriazole-1-yl)-1,1,3,3-tetramethylaminium tetrafluoroborate (TBTU) as the coupling agent in 52% yield over two steps. Finally, the removal of the methyl ester by hydrolysis afforded the new desired derivative UniPR1449 in 78% yield.

**Figure 9:**
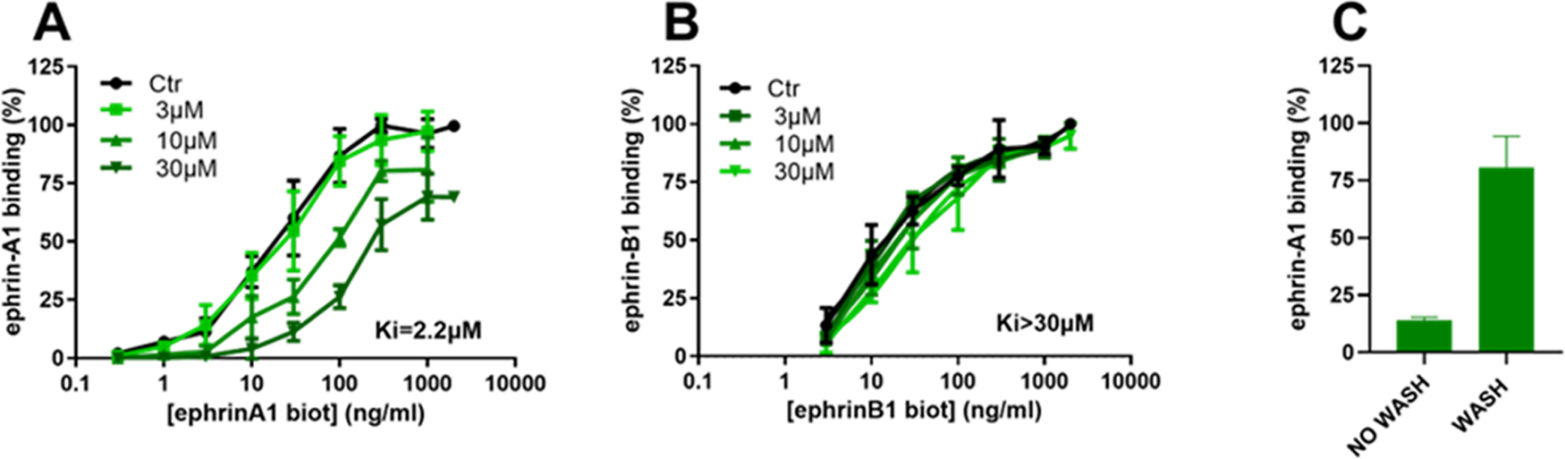
Binding of biotinylated ephrin-A1-Fc to immobilized EphA2 (A) and of biotinylated ephrin-B1-Fc to immobilized EphB2 (B) in the presence of different concentrations of UniPR1449. EphA2–ephrin-A1 binding in the presence of 50 μM UniPR1449 with or without washing three times with PBS before adding ephrin-A1-Fc (C). Data are the means of at least three independent experiments ± standard deviation.

We evaluated UniPR1449 for its ability to prevent biotinylated ephrin-A1 and ephrin-B2 binding to the EphA2 and EphB2 receptors, respectively. We built saturation curves for EphA2–ephrin-A1 binding as well as of EphB2–ephrin-B1 binding in the presence of increasing concentrations of UniPR1449 (Figure 9) to retrieve *K_i_* values for EphA2 and EphB2 by nonlinear regression analysis. Gathered data indicated that UniPR1449 bound EphA2 with a *K_i_* of 2.2 μM, while it failed to engage EphB2 up to a concentration of 30 μM, as confirmed by EphB2–ephrinB1 saturation curves, which were unaffected by the presence of the target compound in the assay. Finally, the binding of UniPR1449 to the EphA2 receptor was reversible.

**Figure 10:**
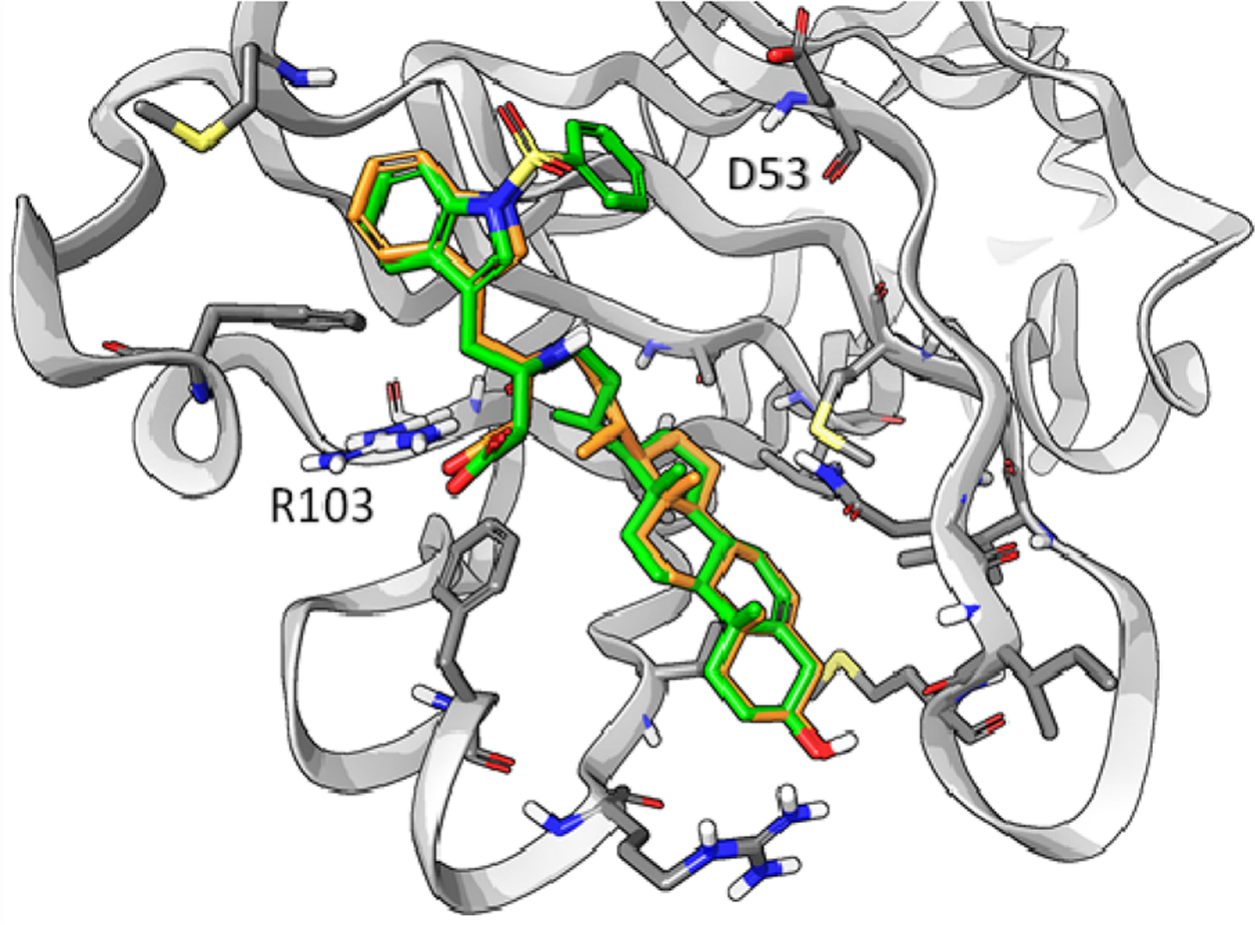
Best pose for UniPR1449 (green carbon atoms) docked within the LBD of EphA2. UniPR1447 (orange carbon atoms) is also reported for comparison.

It thus appears that indole nitrogen substitution at the level of the L-β-homotryptophan moiety might represent a general strategy to obtain selectivity for EphA2 through occupation of an accessory cavity (Figure 10), at least over the EphB2 receptor subtype. This binding model could be exploited for the synthesis of analogues with improved activity and selectivity for the EphA2 receptor.

### Surface Plasmon Resonance on EphA2

**Figure 11:**
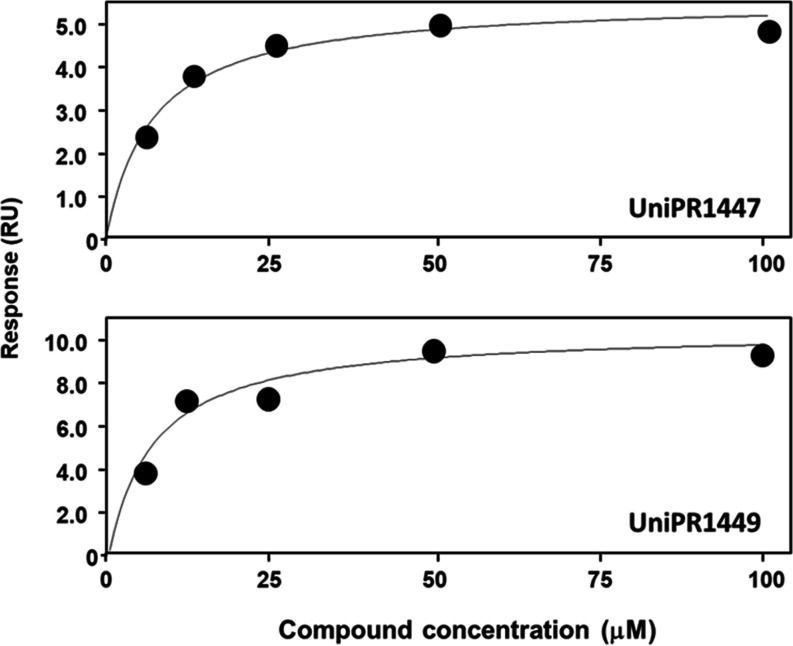
Steady-state analysis of the binding of UniPR1447 and UniPR1449 injected onto the SPR sensorchip containing EphA2. The results shown are representative of five (UniPR1447) and seven (UniPR1449) independent SPR analyses that gave similar results.

Gathered data in the ELISA assay indicate that the binding of ephrin-B1 to EphB2 is prevented by UniPR1447 and not by UniPR1449, while the binding of ephrin-A1 to EphA2 can be prevented by both compounds (Figures 4 and 9). Accordingly, docking and MD simulations predicted that EphA2 would bind the two compounds with similar binding modes (Figure 10). On these bases, we exploited SPR technology to confirm the ability of UniPR1447 and UniPR1449 to target EphA2. Increasing concentrations of the two compounds were injected over EphA2-Fc immobilized on the surface of a CM SPR4 sensorchip and evaluated for their interaction. As shown in Figure 11, when tested under the same experimental conditions, both compounds bound the immobilized EphA2 receptor in a concentration-dependent and saturable manner, confirming that the recognition processes were both specific. The calculated *K*_D_ values were similar for the two compounds, being equal to 3.4 ± 1.7 and 3.8 ± 2.4 μM for UniPR1447 and UniPR1449, respectively, and in line with *K_i_* values measured in the displacement assay based on the ELISA method. Thus, SPR data provided an orthogonal validation of the binding predictions provided by computational studies.

### Physicochemical Properties of UniPR1447 and UniPR1449

We experimentally determined the distribution coefficient (Log* D*_oct_; see the Methods section) for newly synthesized compounds, as this molecular property may affect compound disposition in the proximity of the cell membrane of targeted cells, where Eph receptors and ephrin ligands are expressed. UniPR1447 and UniPR1449 displayed similar Log *D*_oct_ values (4.6 ± 0.10 and 4.7 ± 0.11, respectively), indicating that the introduction of an *N*-sulfonylphenyl substituent did not affect their hydrophilic/hydrophobic balance.

We also evaluated the stability of cell metabolism of both compounds. This was assessed by incubating UniPR1447 or UniPR149 for 60 min at 37 °C with rat plasma and rat liver microsome. In plasma, both compounds were stable with no significant conversion into 3β-hydroxy Δ^5^-cholenic acid and free amino acid. On the other hand, the compounds were subjected to oxidative metabolism in the presence of rat liver microsomes. UniPR1449 (62.2% of the compound recovery at 60 min) was slightly more stable than UniPR1447 (40.3% of the compound recovery), indicating that the presence of the *N*-sulfonylphenyl moiety retarded biotransformations catalyzed by the microsomal fractions either sterically and/or electronically.

### Viability of the U251 Glioblastoma Cell Line

**Figure 12:**
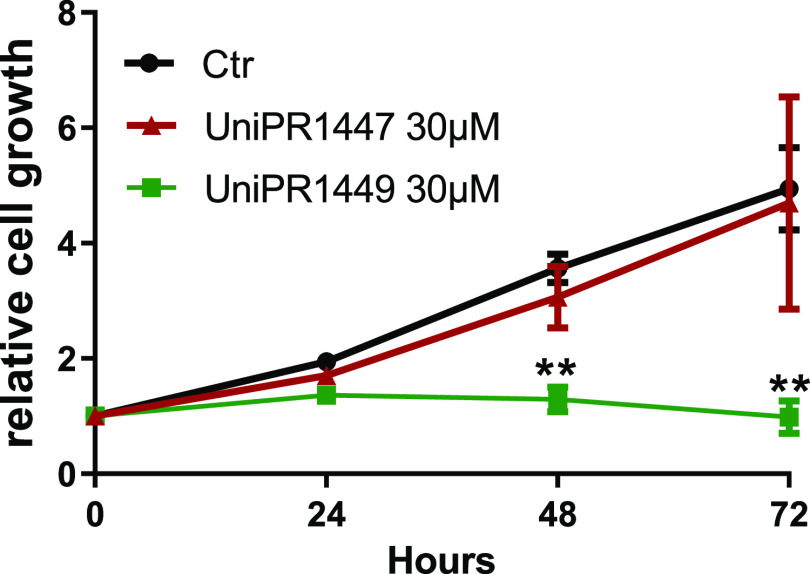
Time course of cell viability on the U251 GBM cell line by Eph–ephrin antagonists UniPR1447 (red line and red triangles) and UniPR1449 (green line and squares). Data are the means of at least three independent experiments ± standard deviation.

The new compounds UniPR1447 and UniPR1449 were tested for their ability to inhibit GBM U251 cell proliferation in an MTT assay. Figure 12 reports the time course of cell growth compared to the control (DMSO 0.3% solution) in the presence of a compound concentration of 30 μM. While UniPR1447 did not significantly affect U251 growth compared to the untreated cells, the continuous exposure to UniPR1449 fully blocked GBM growth already after 48 h. Similar efficacy in growth inhibition was observed at a significantly higher concentration with the reference compound UniPR1331 (100 μM, 72 h) or the approved drug Temozolomide (500 μM, 72 h), as shown in the SI (Figure S10). Although additional investigations will be required to confirm this promising behavior, it could be speculated that the higher antiproliferative activity of UniPR1449 compared to that of UniPR447, and the reference antagonist UniPR1331, is due to its peculiar pharmacological profile at the level of Eph receptors.

## Conclusions

Microsecond-long MD simulations allowed the conformational free-energy of binding of the nonselective ligand UniPR1447 in the presence of the EphA2 or EphB2 receptor. Simulations pointed to the presence of an accessory area in the EphA2 receptor, exposed to the solvent, that in EphB2 was occupied by the acid residue Glu52. This difference prompted us to introduce a bulky substituent at the nitrogen atom of the indole ring of UniPR1447 with the final aim of achieving selectivity for the EphA2 receptor. Experimental data supporting our computational hypothesis led to the discovery of UniPR1449, a new compound displaying low micromolar affinity for EphA2 unaffecting EphB2–ephrin-B1 binding up to 30 μM. The new compound, UniPR1449, displayed fair physicochemical properties and was able to inhibit growth of GBM cells more potently than the reference pan Eph–ephrin antagonist UniPR1331. The present work paves the road to a new class of selective agents targeting the Eph–ephrin system.

## Methods

### Chemistry

General information and details for each synthesized compound are given in the Supporting Appendix. Final compounds and key intermediates were characterized by ^1^H- and ^13^C-NMR analyses. The exact mass for final compounds was measured by high-resolution mass spectrometry (HR-MS) following the procedure described in the SI. Optical rotation power for final compounds was derived from ECD spectra (see the SI for details). The purity of each compound was assessed by HPLC/MS analysis and was shown to be 95% or higher. ^1^H-NMR, ^13^C-NMR, and HR-MS spectra are provided in the SI section.

### Molecular Modeling

#### Protein Preparation

The crystallographic structures of the EphA2–ephrin-A1 (PDB ID: 3HEI, human protein) and of the EphB2–ephrin-B2 (PDB ID: 1KGY, murine protein) complex were prepared using the Protein Preparation Wizard Tool of the Schrödinger 2021–2 suite.^49^ Human and murine EphB2 share 99.4% identity, with no mutations occurring at the level of the ligand binding domain. The EphB2 receptor was therefore not modified in its sequence. In both EphA2–ephrin-A1 and EphB2–ephrin-B2 complexes, missing hydrogen atoms were added, and orientation of hydroxyl groups and conformations of asparagine and glutamine side chains were modeled to maximize the number of hydrogen bonds. Acid (Asp and Glu) and basic residues (Arg and Lys) were modeled in the charged form. The resulting structures were then submitted to a restrained minimization step with the OPLS4 force field,^50^ during which only the hydrogen atoms were free to move. This step was followed by a second minimization restrained to a root-mean-square deviation (RMSD) of 0.3 Å calculated for the heavy atoms. Before docking simulations, the water molecules and the ephrin-A1 or ephrin-B2 molecules were removed.

#### Docking Simulations

Docking studies were performed using Glide version 9.1.^51^ The docking grids were placed in the hydrophobic channel of EphA2 and EphB2 that accommodates the G-H loop of the ephrin. The grids were centered on the center of mass of residues Arg103, Phe108, and Phe156, in the case of EphA2, and on the center of mass of residues Arg103, Ile108, and Phe155, in the case of EphB2. The dimensions of the enclosing and bounding boxes were set to 20 and 10 Å on each side, respectively. Default settings were applied, and the van der Waals radii of the protein were not scaled. The three-dimensional structures of UniPR1447 and UniPR1449 were built in Maestro 12.8 and optimized through an energy minimization step with Macromodel^52^ applying the OPLS4 force field in an implicit water model,^53^ using the Polak–Ribiere conjugate gradient method^54^ to an energy gradient of 0.01 kj/mol/Å. The energy-minimized structures were then used in docking simulations, performed in the standard precision (SP) mode, to generate the UniPR1447–EphA2/EphB2 and UniPR1449–EphA2 complexes. The resulting docking poses were collected and ranked according to their G_score_ value.

#### MD Simulations of the UniPR1447–EphA2/EphB2 Complexes

The UniPR1447–EphA2 and UniPR1447–EphB2 complexes were solvated by approximately 12000 TIP3P water molecules and neutralized by adding 5 and 2 Na^+^ ions, respectively, setting the orthorhombic box dimensions to 10 Å. The resulting systems were submitted to MD simulations with the OPLS4 force field using a Desmond 6.6 engine^55^ on NVDIA GeForce RTX 3070 graphic cards. The systems were equilibrated for 13 ns, during which the temperature of the systems was progressively increased to 300 K, and the constraints on the heavy atoms of the ligand and the proteins were gradually reduced. Bond lengths to hydrogen atoms were constrained by applying the M-SHAKE algorithm. Short-range electrostatic interactions were cut off at 9 Å, whereas long-range electrostatic interactions were treated using the smooth particle mesh Ewald method (PME).^56^ A RESPA integrator was used with a time step of 2 fs, while long-range electrostatic interactions were computed every 6 fs, similarly to what was performed on other protein–ligand systems.^57^ The production was carried out for 2.5 μs in the canonical ensemble using the Langevin thermostat. During the simulations, harmonic restraints of 0.5 and 1 kcal/mol/Å^2^ were applied on the Cα atoms of the protein and, on the heavy atoms of the steroidal scaffold, the amide and carboxylic group of the ligand, respectively. To investigate the conformations explored by the tryptophan portion limited to the side-chain torsion, two variables, χ1 and χ2, describing the dihedrals formed by the N–Cα-Cβ-Cγ and Cα-Cβ-Cγ-Cδ1 atoms of the tryptophan, were monitored throughout the simulations, while a harmonic restraint of 10 kcal/mol/rad^2^ was applied to the φ angle. For both the UniPR1447–EphA2 and UniPR1447–EphB2 systems, three 2.5 replicas were performed by setting different initial velocities and setting the random number generator seed values to 2007, 221212, and 8902, respectively.

#### MD Simulation Analysis

The trajectories collected for each MD simulation were analyzed, and the values measured for the χ1 and χ2 variables were used to calculate the free-energy of the systems by applying the Boltzmann distribution. The conformational space described by the two variables, spanning from −180 to 180°, was divided into histograms (each covering 15°), and the occurrence of each state was computed. The free-energy was finally calculated using Microsoft Excel by applying the following equation:
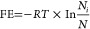
where *RT* is the Boltzmann constant at *T* = 300 K, *N*_*i*_ is the number of frames representing a specific state, and *N* is the total number of frames collected for each MD simulation (25,000 for both systems). The convergence was assessed by evaluating the evolution of the FES during MD simulations. Simulations were considered converged after 1 μs, as no significant variations occurred between minima in terms of position on the FESs and of relative free-energy for both EphA2 and EphB2 systems (see Figures S5, S6, S8 and S9 in the SI), similarly to what was performed in our previous works on conformational free-energy.^58^

### Biological Assays

#### Reagents

All culture media and supplements were bought from Euroclone (Milano, Italy). Recombinant proteins and antibodies were from R&D System (Minneapolis, Minnesota). Leupeptin, aprotinin, NP40, tween20, BSA, and salts for solution were from ITW Reagents (Chicago, Illinois); EDTA and sodium orthovanadate were from Merk (Darmstadt, Germany).

#### ELISA Displacement Assay and *K_i_*/IC_50_ Determination

96-well ELISA high binding plates (Corning Costar, 9018) were incubated overnight at 4 °C with 100 μL/well of 1 μg/mL EphA2-Fc (R&D System, 639-A2) diluted in sterile phosphate-buffered saline (PBS, 0.2 g/L KCl, 8.0 g/L NaCl, 0.2 g/L KH2PO4, 1.65 g/L Na2HPO4, pH 7.4). The day after, wells were washed with a washing buffer (PBS 0.05% tween20, pH 7.5) and blocked with a blocking buffer solution (PBS 1% BSA) for 1 h at 37 °C. Compounds were added to the wells at a proper concentration in 0.5% DMSO and incubated at 37 °C for 1 h. Biotinylated ephrin-A1-Fc (R&D Systems, BT602) was added at 37 °C for 4 h at its *K*_D_ value in displacement assays or in a range from 1 to 2000 ng/mL in saturation studies. After 4 h for displacement assays or 5 h for saturation studies, wells were washed and incubated with 100 μL/well Streptavidin-HRP (Sigma S5512) for 20 min at room temperature. Then, wells were washed again with a washing buffer and incubated at room temperature with 0.1 mg/mL tetramethylbenzidine (Sigma T2885) reconstituted in stable peroxide buffer (0,05 M citric acid +0,05 M dibasic sodium phosphate, pH 5). The reaction was quenched with 3 N HCl. Absorbance was read at 450 nm (ELISA plate reader). The IC_50_ and *K_i_* values were determined using one-site competition nonlinear regression analysis with Prism software (GraphPad Software Inc.).

#### Surface Plasmon Resonance

Surface plasmon resonance (SPR) measurements were performed on a BIAcore X100 instrument, using a carboxyl-methyl-dextran-coated sensorchip (CM4, GE-Healthcare). SPR was employed to measure changes in the refractive index caused by the binding of UniPR1447 and UniPR1449 to surface-immobilized immobilized EphA2. To this aim, the fusion protein EphA2-Fc (RnD Systems, 639-A2–200) or the Fc fragment alone (Millipore, AG714) (here used as a negative control) was resuspended at 20 μg/mL in 10 mM sodium acetate pH 4.0 and allowed to react with two separate flow cells of a CM4 sensorchip, preactivated with 50 mL of 0.2 M *N*-ethyl-*N*-(3-diethylaminopropyl)carbodiimide hydrochloride and 0.05 M *N*-hydroxysuccinimide, leading to the immobilization of 3000 and 950 RU for EphA2-Fc and Fc fragments, respectively (equal to approximately 40 fmol/mm2 for both proteins). Increasing concentrations of UniPR1447 or UniPR1449 in PBS, 0.05% surfactant P20, and 5% DMSO, pH 7.4 were injected over the EphA2 or Fc surfaces for 90 s and then washed until dissociation was observed. Dissociation constant (*K*_D_) values were measured from steady-state analysis obtained by plotting the relative binding at equilibrium vs the ligand concentration and considering the *K*_D_ equal to the concentration yielding 50% of the maximum response. The K_D_ values ± standard deviation reported above were obtained from five and seven measurements for UniPR1447 and UniPR1449, respectively.

#### Log *D*_oct,7.4_ Measurements

Distribution coefficients (*D*) were measured at physiological pH by the shake-flask method at room temperature (21.0 ± 0.5 °C), employing *n*-octanol/50 mM MOPS buffer pH 7.4, 0.15 M KCl ionic strength as a biphasic solvent system, after mutual saturation by overnight stirring. A weighed amount of compound was dissolved in water-saturated n-octanol, a buffer was added, and the sample was stirred for 4 h to reach partition equilibrium. Partition phases were centrifuged (9000*g*, 10 min, 20 °C), separated, diluted with methanol, and dosed by HPLC-ESI-MS/MS.

#### Metabolic Stability Assays

Experiments were performed as previously described.^59^ Briefly, stock solutions of UniPR1447 and UniPR1449 were prepared in DMSO immediately before use. Pooled plasma (400 μL) was incubated at 37 °C in the presence of 10 mM phosphate-buffered saline (PBS) pH 7.4. 5 μL of compound stock solution in DMSO (final DMSO concentration: 1%; final compound concentration: 1 μM) was added to start the rat plasma stability assay. In the rat liver microsome stability assay, aliquots of rat liver microsomes were activated by a 5 min incubation at 37 °C with an NADPH-generating system (2 mM NADP^+^, 10 mM glucose-6-phosphate, 0.4 U/mL glucose-6-phosphate dehydrogenase, 5 mM MgCl_2_) in PBS pH 7.4. At the end of preincubation, a compound stock solution (5 μL, 100 μM) in DMSO was added (final DMSO concentration in samples: 1%; final compound concentration: 1 μM). At fixed time points, aliquots of 50 μL were taken, added with two volumes of CH_3_CN containing as internal standard the structural analogue UniPR126, centrifuged (16,000*g*, 5 min, 4 °C), and analyzed by HPLC-ESI-MS/MS. The responses (i.e., peak area ratios of test compound vs internal standard) were referred to the zero time-point samples (considered as 100%) to determine the percentage of remaining compound vs time.

## Data Availability

Molecular structures, input files used to run the simulations are made available in the Supporting Information. The Schrödinger Suite 2021 used in this work is distributed under license (https://www.schrodinger.com).

## Supporting Information Available

Superposition of X-ray structures of EphA2 and EphB2, analysis of evolution of χ1 and χ2 throughout MD simulation, analysis of convergence for the three replicas for the EphA2– and EphB2–UniPR1447 complexes, geometries of an alternative minimum on the FES for EphA2–UniPR1447 complex, viability of the U251 glioblastoma cell line treated with UniPR1331 and Temozolomide, syntheses of UniPR1447 and UniPR1449, ^1^H- and ^13^C-NMR analyses for intermediates and final compounds, and HPLC/MS analysis (DOCX)
Binding modes used for MD simulations for the compounds under investigation, input files and geometries used to run MD simulations with Desmond 6.6 implemented in Schrödinger 2021–2 (ZIP)

## Abbreviations

ELISA: enzyme-linked immunosorbent assay
EphA2: ephrin receptor A2
EphB2: ephrin receptor B2
MD: molecular dynamics
FES: free-energy surface
SPR: surface plasmon resonance
