# Supplementary material for: Molecular Determinants of EphA2 and EphB2 Antagonism Enable the Design of Ligands with Improved Selectivity
Source: J Chem Inf Model. 2023 Nov 1;63(21):6900–11. doi: 10.1021/acs.jcim.3c01064 (PMC10647059; doi:10.1021/acs.jcim.3c01064)
Supplement: Supplementary file 1 — ci3c01064_si_001.docx [file ci3c01064_si_001.docx]

Molecular Determinants of EphA2 and EphB2 Antagonism Enable the Design of Ligands with Improved Selectivity.

Lorenzo Guidetti, ^‡a^ Alfonso Zappia, ^‡ a^ Laura Scalvini,^a^ Francesca Romana Ferrari,^a^ Carmine Giorgio, ^a^ Riccardo Castelli,^a^ Francesca Galvani,^a^ Federica Vacondio,^a^ Marco Mor,^a,b^ Chiara Urbinati,^c^ Marco Rusnati,^c^ Massimiliano Tognolini^* a^ and Alessio Lodola ^* a^

^a^ Dipartimento di Scienze degli Alimenti e del Farmaco, Università degli Studi di Parma, Parco Area delle Scienze 27/A, I- 43124 Parma, Italy.

^b^ Microbiome Research Hub, Università degli Studi di Parma, Parco Area delle scienze 11/A, I- 43124 Parma, Italy.

^c^ Dipartimento di Medicina Molecolare Traslazionale, Università degli Studi di Brescia, Brescia, Italy

AUTHOR INFORMATION

Corresponding Author

* Phone: +39 0521 905062. Fax: + 39 0521 905006. E-mail: [alessio.lodola@unipr.it](mailto:alessio.lodola@unipr.it)

* Phone: +39 0521 906021. Fax: + 39 0521 905006. E-mail: [massimiliano.tognolini@unipr.it](mailto:massimiliano.tognolini@unipr.it)

# Dose-response curve in displacement assay for UniPR1447


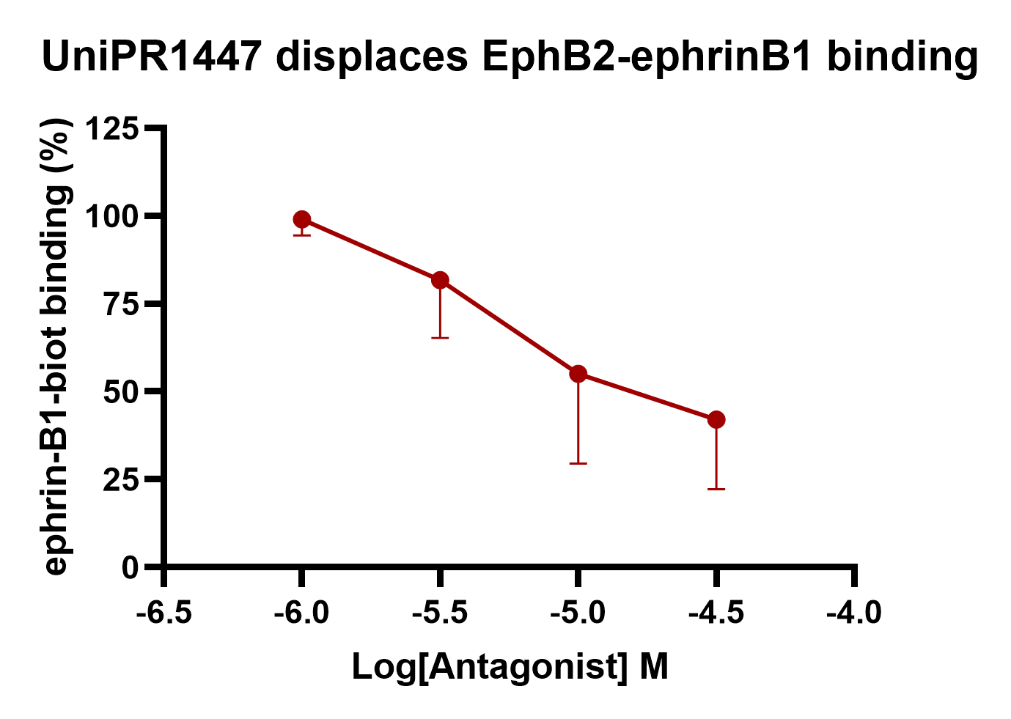


**Figure S1**. Dose-response curve for UniPR1447 in displacement assay of biotinylated ephrin-B1-Fc from EphB2-Fc.

# Superposition of EphA2 and EphB2 X-ray structures


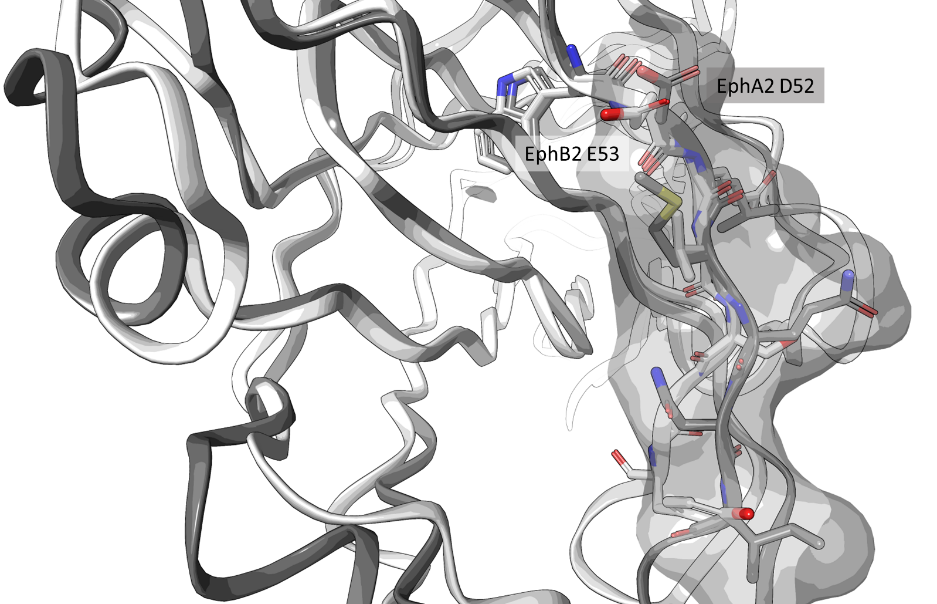


**Figure S2.** Superposition of the X-ray structures of EphA2 (gray carbon atoms, cartoon and surface; PDB ID: 3HEI) and EphB2 (white carbon atoms and cartoon; PDB ID: 1KGY). Residues on β-strand C are represented in sticks. The replacement of Asp52 (EphA2) with Glu53 (EphB2) accounts for a reduced volume of this region of the ligand binding domain of EphB2.

# Temporal evolution of χ1 and χ2 dihedral angles for EphA2-UniPR1447 complex.


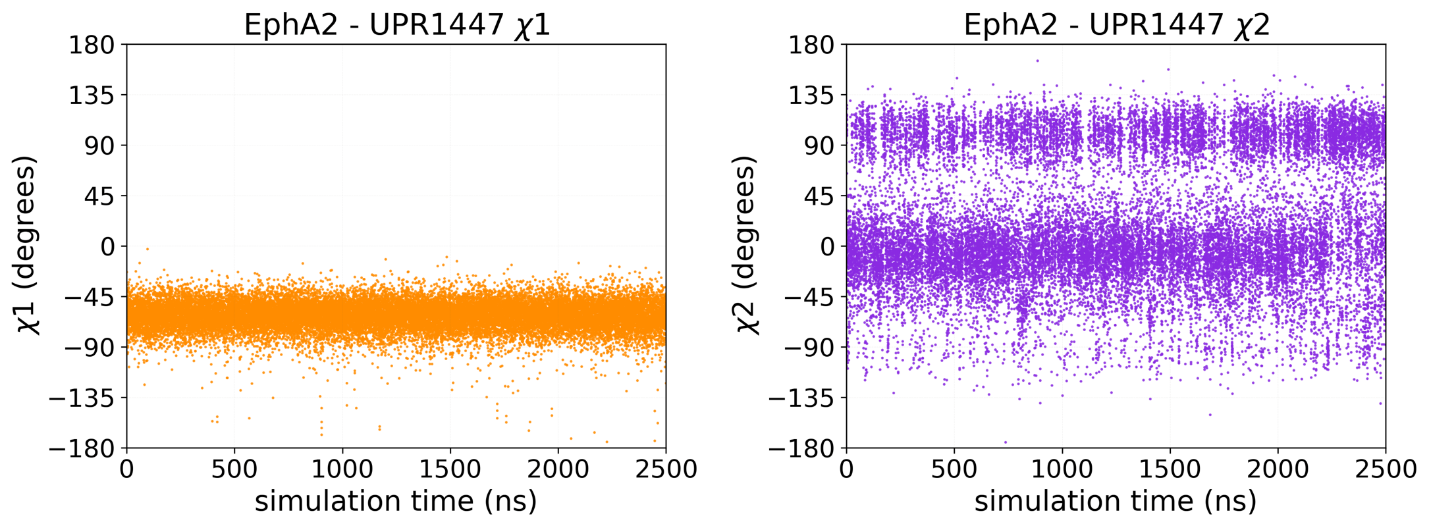


**Figure S3**. Temporal evolution of dihedral angles χ1 (described by atoms N-Cα-Cβ-Cγ, left panel, orange points) and χ2 (described by atoms Cα-Cβ-Cγ-Cδ1, right panel, purple points) during a 2.5μs MD simulation for EphA2-UniPR1447 complex.

# Conformation corresponding to an alternative minimum on the FES for EphA2-UniPR1447 complex.


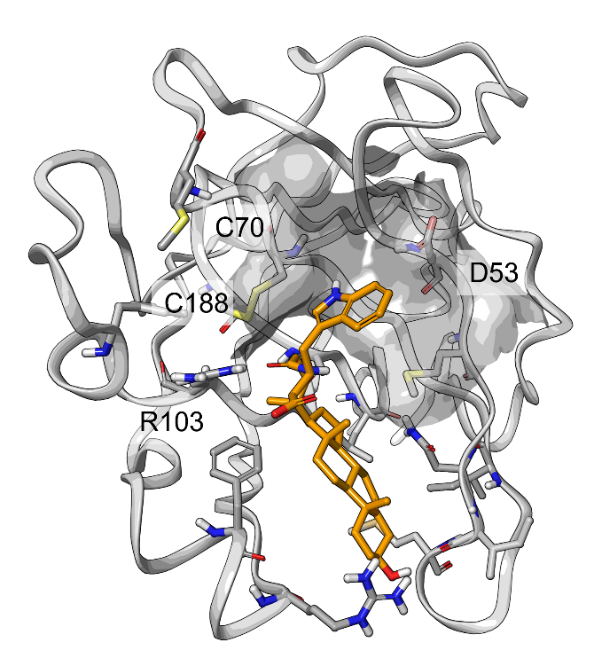


**Figure S4.** Geometries corresponding to the second minimum identified on the FES of EphA2-UniPR1447 complex (grey and orange carbon atoms, respectively). χ1 and χ2 assumed values corresponding to -60° and 90°, with the -NH group of the indole pointing towards a lipophilic region of the LBD delimited by the disulfide bridge between Cys70 and Cys188 (represented as grey surface).

# Analysis of convergency of FES for EphA2-UniPR1447 complex.


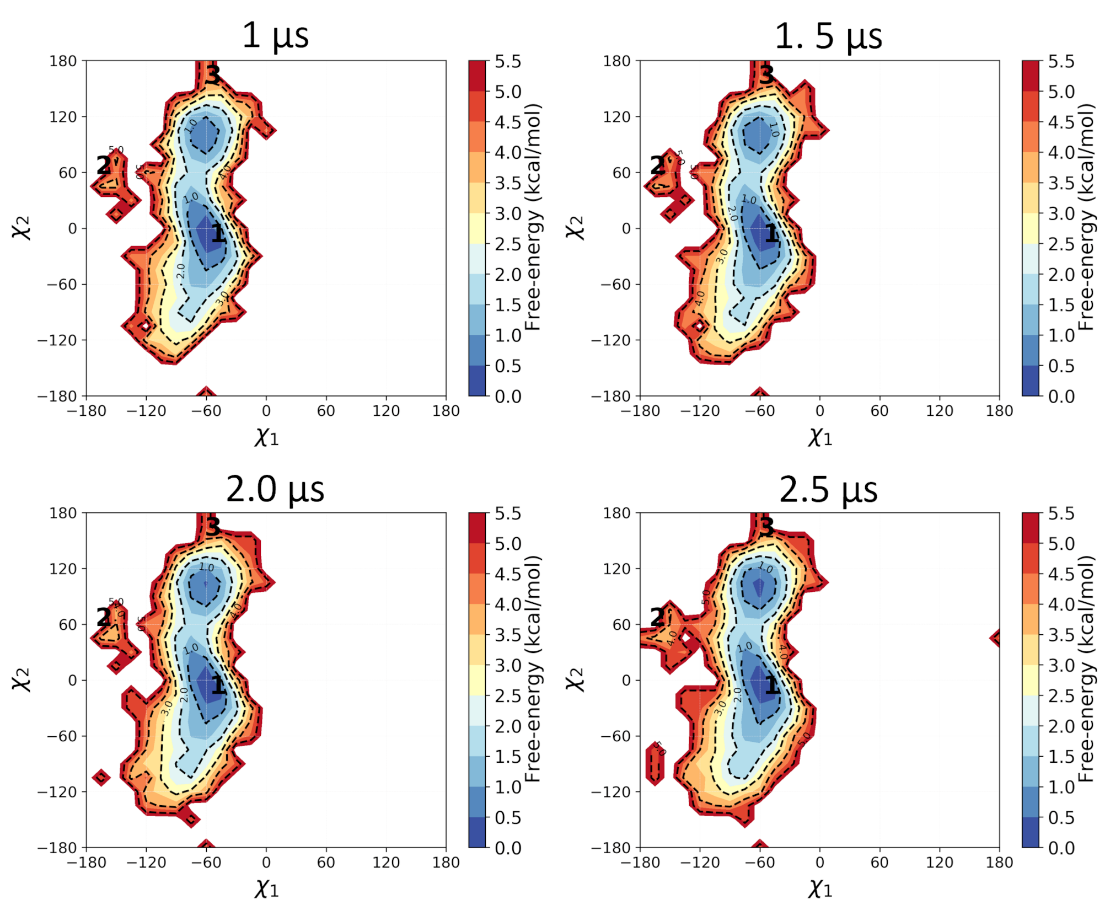


**Figure S5.** Analysis of convergency of FES for EphA2-UniPR1447 complex along the dihedrals χ1 and χ2. FESs were reconstructed at 1μs, 1.5μs, 2μs and 2.5μs. The simulation appeared converged after 1μs, as the FES did not show any variation in both position and relative energies of the minima.

# FESs reconstructed from 2.5μs replicas of EphA2-UniPR1447 MD simulation.


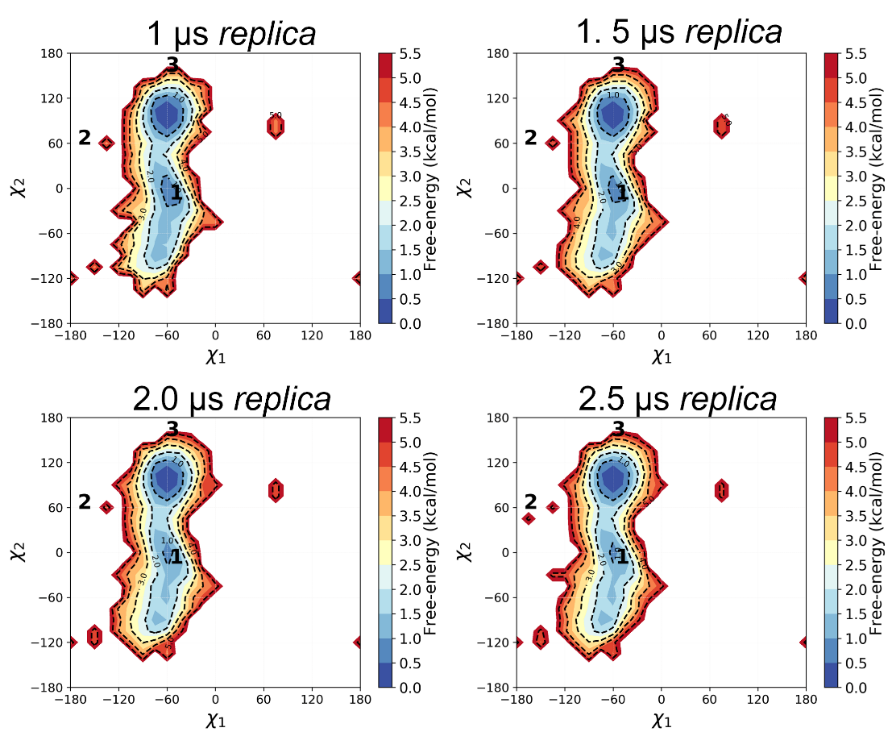


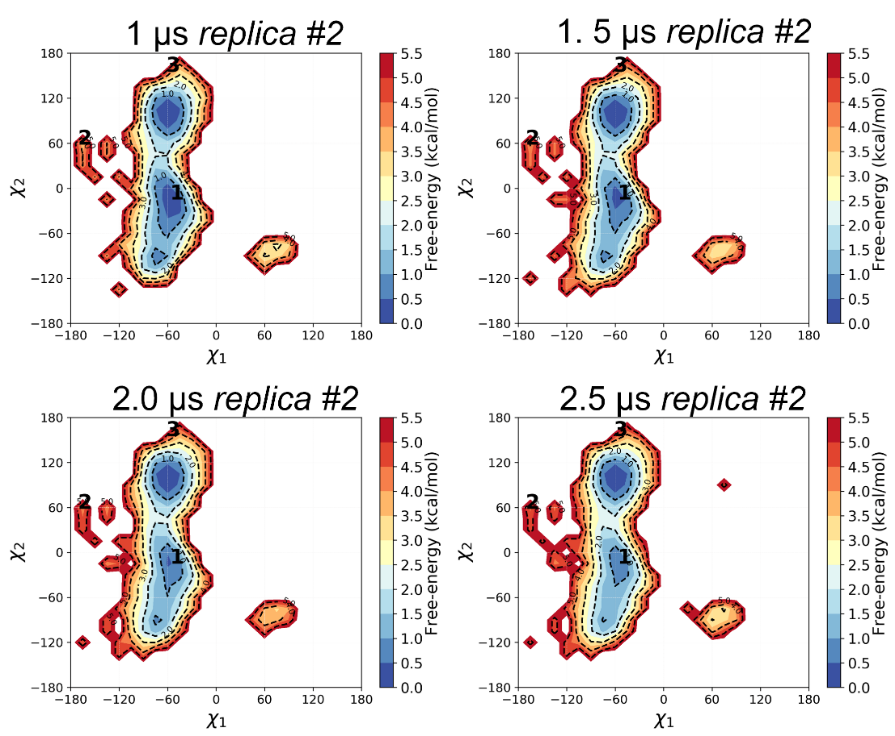


**Figure S6.** FESs reconstructed from two different 2.5μs replicas of EphA2-UniPR1447 MD simulation along the dihedrals χ1 and χ2. The FESs were reconstructed at 1μs, 1.5μs, 2μs and 2.5μs.

# Temporal evolution of χ1 and χ2 dihedral angles for EphB2-UniPR1447 complex.


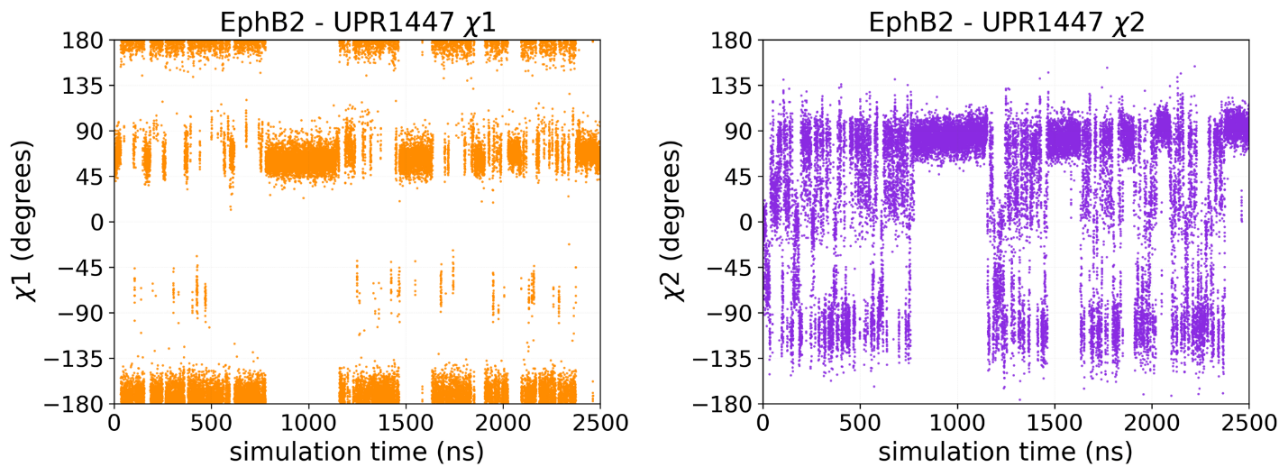


**Figure S7**. Temporal evolution of dihedral angles χ1 (described by atoms N-Cα-Cβ-Cγ, left panel, orange points) and χ2 (described by atoms Cα-Cβ-Cγ-Cδ1, right panel, purple points) during a 2.5μs MD simulation for EphB2-UniPR1447 complex.

# Analysis of convergency of FES for EphB2-UniPR1447 complex.


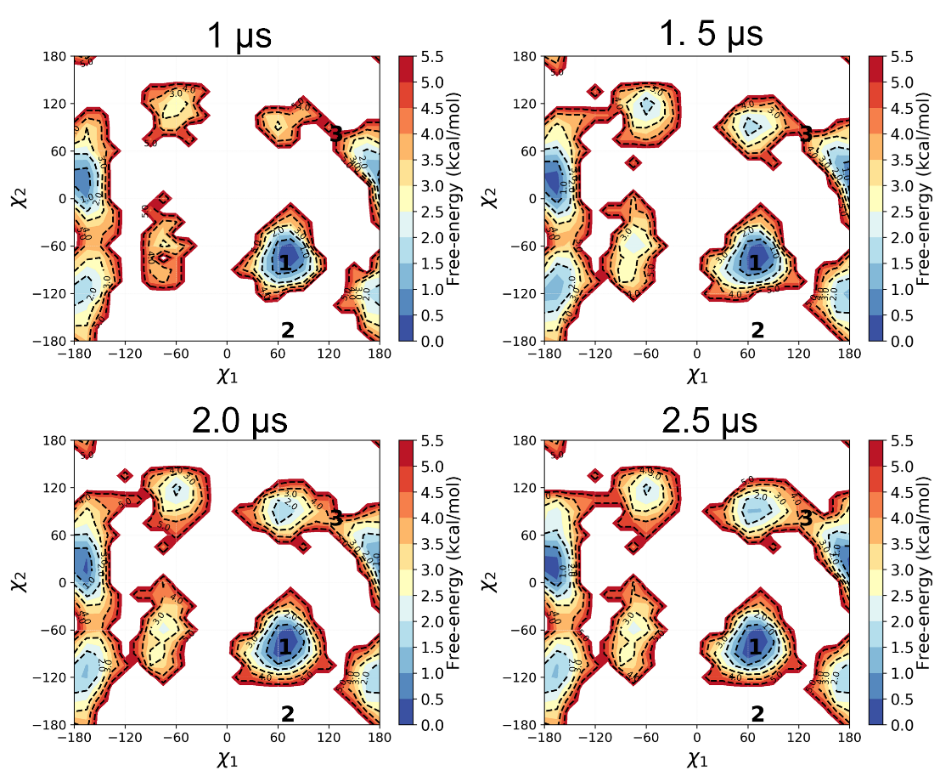


**Figure S8.** Analysis of convergency of FES for EphB2-UniPR1447 complex along the dihedrals χ1 and χ2. FESs were reconstructed at 1μs, 1.5μs, 2μs and 2.5μs. The simulation appeared converged after 1μs, as the FES did not show any variation in both position and relative energies of the minima.

# FESs reconstructed from 2.5μs replicas of EphB2-UniPR1447 MD simulation.


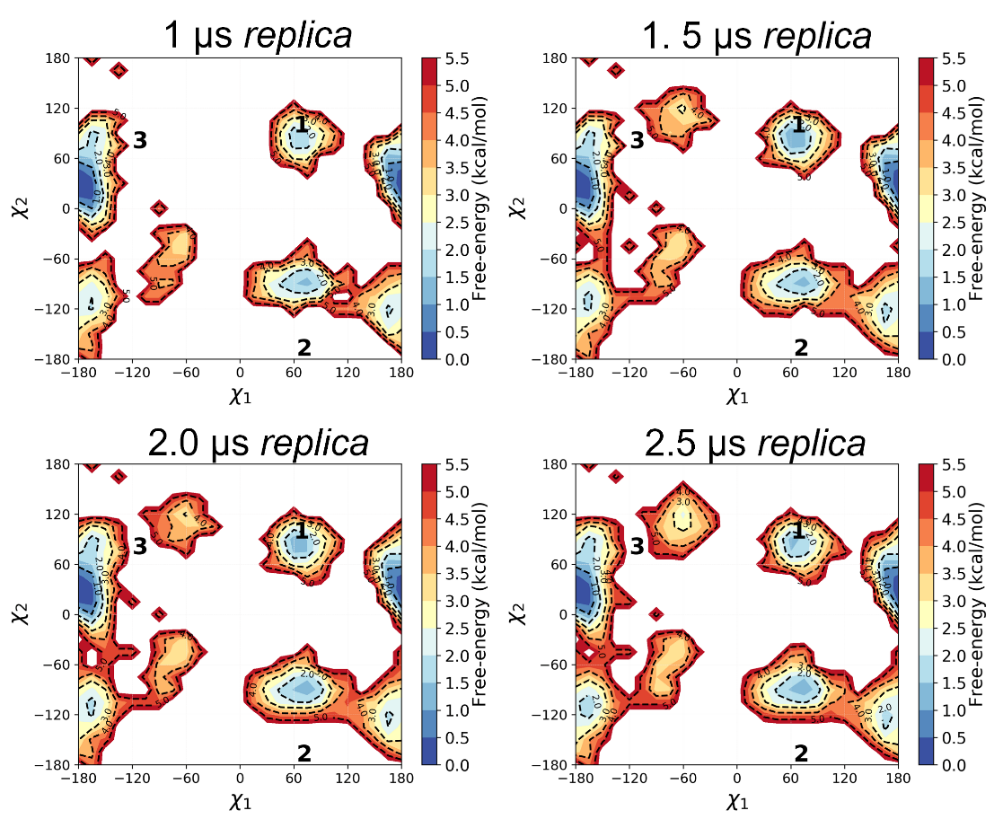


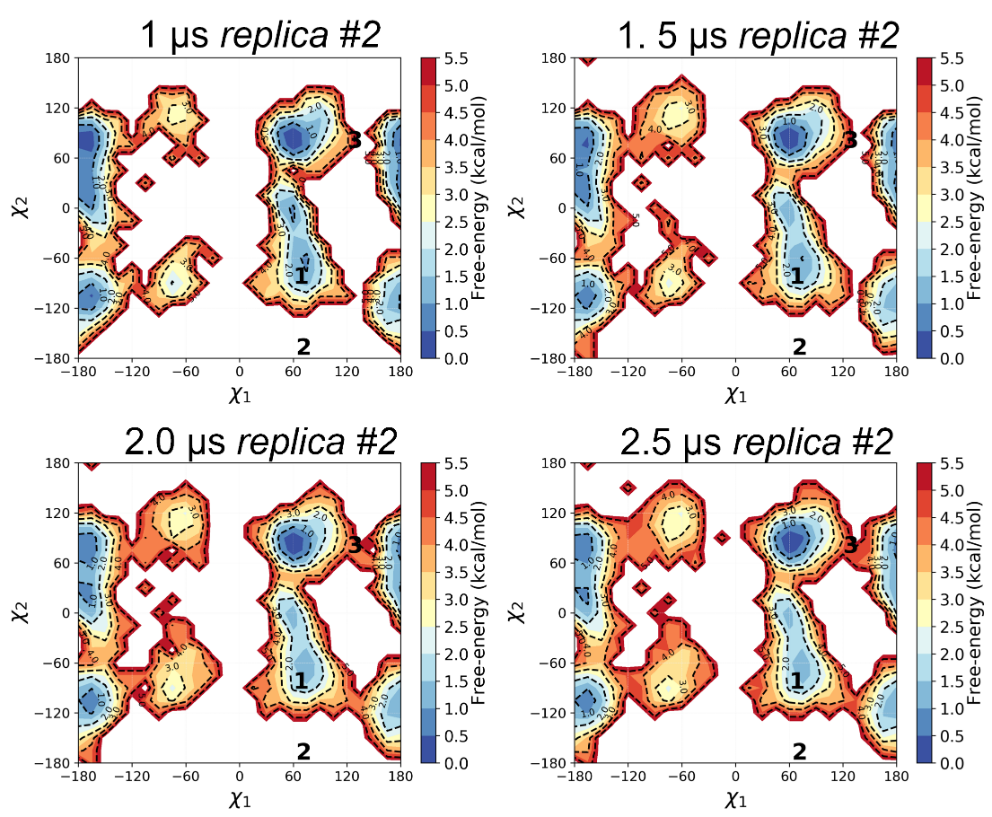


**Figure S9.** FESs reconstructed from two different 2.5μs replicas of EphB2-UniPR1447 MD simulation along the dihedrals χ1 and χ2. The FESs are calculated at 1μs, 1.5μs, 2μs and 2.5μs.

# Cell viability assay

**Figure S10.** Cell viability assay performed using a MTT assay on U251 glioblastoma cell line treated with the approved drug temozolomide and the reference compound UniPR1331.

# Chemistry

# Synthesis ad characterization of final compounds

All chemicals were used as received unless stated otherwise. All reactions were performed under a steady overpressure of nitrogen delivered through a balloon. Anhydrous solvents such as dichloromethane, *N,N*-dimethylformamide and toluene were directly purchased from an appropriate vendor. Column chromatography was performed on silica gel 60 (0.040-0.063 mm) under forced flow of the appropriate solvent mixtures. TLC analysis were conducted on HPTLC aluminum sheets (Sigma-Aldrich, silica gel 60, F_254_), compounds were visualized by UV absorption (245 nm) and/or by dipping in a solution of (NH_4_)_6_Mo_7_O_24_·4 H_2_O 25 g/l and (NH_4_)_4_Ce(SO_4_)_4_·2 H_2_O 10 g/l, in 1 L of 10% aqueous H_2_SO_4_ (CAM solution). Melting points were determined with a Gallenkamp melting point apparatus.

**NMR data (all synthesized compounds).** The ^1^H-NMR and ^13^C-NMR spectra were recorded on a Bruker Avance 400 spectrometer (400 MHz) or a JEOL ECZ600R (600 MHz); chemical shifts (δ scale) are reported in parts per million (ppm). ^1^H-NMR spectra are reported in the following order: multiplicity, approximate coupling constants (*J* value) in Hertz (Hz) and number of protons; signals were characterized as s (singlet), d (doublet), t (triplet), q (quartet), m (multiplet), bs (broad signal).

**HR-MS data acquisition for compounds UniPR1447 and UniPR1449.** A Thermo Exploris 120 high resolution mass spectrometer interfaced with a Thermo Vanquish Flex Ultra high-performance chromatography system (Thermo, USA) was employed for HR-MS data acquisition for compounds **UniPR1447** and **UniPR1449**. A Waters HSS T3 column (2.1 x 100 mm, 1.7 μm particle size; Waters, USA) was employed for gradient elution of compounds. Eluent A was water; eluent B was acetonitrile. Both phases were acidified with 0.1% v/v HCOOH. Gradient was as follows: t=0 min: 80%A; t=6 min: 5%A; t=8 min: 5%A; t=9 min:80%A followed by a reconditioning time of 3 min. Total run time: 12 min. Flow rate: 0.30 mL/min; injection volume: 5 μL. HR-MS scans were performed in Full Scan mode (m/z = 100-1000 amu) and in positive electrospray (ESI+). Capillary temperature: 325 °C; sheath gas pressure: 50 (arbitrary unit, a.u.); auxiliary gas pressure: 10 a.u.; sweep gas pressure: 1 a.u.; vaporizer temperature: 350°C; ESI source voltage was set at 3500 V; Resolution was set at 30000.

**Optical rotatory power for compounds UniPR1447 and UniPR1449.** Optical rotation data [α]D were obtained through ECD spectra using Jasco J715 spectropolarimeter at room temperature using a 100 mm cell with 1- or 5-mL capacity and are given in units of 10-1 deg·cm2·g-1.

**Purity assessment of UniPR compounds.** An Accela UHPLC system (Thermo, USA) interfaced with a TSQ Quantum Access Max Triple Quadrupole mass spectrometer (Thermo, USA) with a heated electrospray (H-ESI) ion source was employed for purity assessment of UniPR compounds. Chromatographic separation occurred on a Waters Xselect HSS T3 column (2.1 X 100 mm, 3.5 mm particle size) in gradient mode. Eluent A: acetonitrile + 0.1% v/v formic acid; eluent B: ultra-pure water + 0.1% v/v formic acid. T=0 min: 5%A; 0-12 min: 5-95%A; 12-16 min: 95%A; 16-17 min: 95-5%A; 17-20 min: 5%A. Total run time: 20 min. Flow rate: 0.22 mL/min; injection volume: 10 µL. Mass spectrometer operated in positive ion (ESI+) and in full scan mode (*m/z* = 200-800 amu). The following tune parameters were employed: ion source voltage: 4000 V; Capillary temperature: 270 °C; sheath gas (N_2_): 35 arbitrary units (a.u.); auxiliary gas (N_2_): 15 a.u.; collision gas (Ar) pressure: 1.5 mtorr. Xcalibur software version 2.2 (Thermo, USA) was employed for both data acquisition and processing.

**UniPR1447**

***N*-(3β-hydroxy-Δ^5^-cholen-24-oyl)-L-β-homotryptophan (UniPR1447)**.

To a stirred solution of cholenic acid (200 mg, 0.53 mmol,1.2 eq.) in DMF (10.0 mL) at 0 °C, TBTU (*O*-(Benzotriazol-1-yl)-*N*,*N*,*N′*,*N′*,-tetramethyluronium tetrafluoroborate, 173 mg, 0.53 mmol, 1.2 eq.) is added, followed by DIPEA (*N*,*N*-diisopropyl-*N*-ethylamine, 380 μL, 2.2 mmol, 5eq.) at 0 °C. When a perfectly clear solution is obtained, L-β-Homotryptophan hydrochloride (112 mg, 0.44 mmol, 1 eq.) is added and the mixture is stirred for 16 hours. The mixture is poured over AcOEt (50 mL) and neutralized. The aqueous layer is discarded, and the organic layer is washed with brine. The organic phase is dried over Na_2_SO_4_ and evaporated under reduced pressure. The crude material thus obtained is purified by silica gel column chromatography eluting at first with a 5% solution of methanol in dichloromethane, then with a mixture of DCM:MeOH:AcOH = 95:5:0.5 v/v/v to obtain **UniPR1447** (200 mg, yield = 65%). M.p: 129° C with decomposition. ^1^H NMR (CD_3_OD/CDCl_3_ 1/1, 400 MHz) δ 7.61 (d, *J* = 7.9 Hz, 1H), 7.35 (d, *J* = 8.0 Hz, 1H), 7.16 – 7.09 (td, *J* = 7.3, 0.8 Hz, 1H), 7.08 – 7.01 (m, 2H), 5.36 – 5.28 (m, 1H), 3.39 – 3.38 (m, 1H), 3.12 – 2.90 (m, 2H), 2.62 – 2.39 (m, 2H), 2.32 – 2.09 (m, 3H), 2.04 – 1.89 (m, 4H), 1.88 – 1.73 (m, 3H), 1.72 – 1.63 (m, 1H), 1.63 – 1.32 (m, 8H), 1.31 – 1.01 (m, 7H), 0.99 (s, 3H), 0.90 (d, *J* = 6.5 Hz, 3H), 0.66 (s, 3H). ^13^C {^1^H} NMR (CD_3_OD /CDCl_3_ 1/1, 100 MHz) δ 174.57, 174.31, 140.82, 136.45, 127.63, 123.20, 121.37, 118.79, 118.38, 111.21, 110.65, 71.17, 56.70, 55.73, 50.11, 47.08, 42.27, 41.71, 39.70, 37.66, 37.21, 36.41, 35.47, 33.41, 31.84, 31.77, 31.00, 29.43, 27.94, 24.11, 20.95, 19.09, 18.03, 11.55. ESI(+)-MS calc. for [C_36_H_50_N_2_O_4_+H]^+^: 575.3843; found: 575.3838; Error: -0.87 ppm. [α]_D_^25^ = -16.2° (c = 0.019 g/100 ml, MeOH).


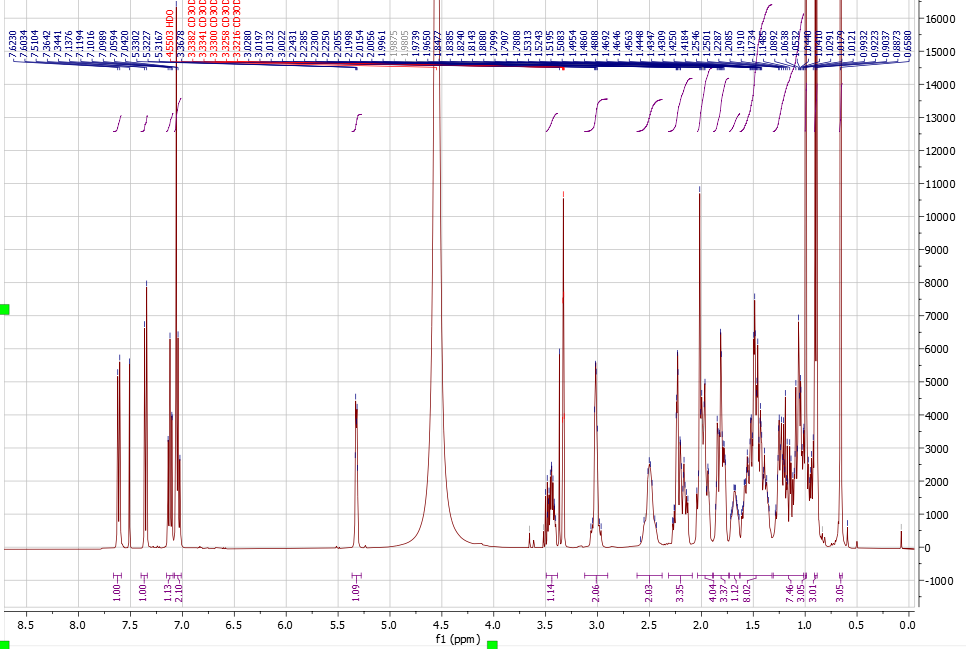


CDCl_3_

CD_3_OD

^1^H NMR (CD_3_OD/CDCl_3_,1/1 400 MHz) **UniPR1447**


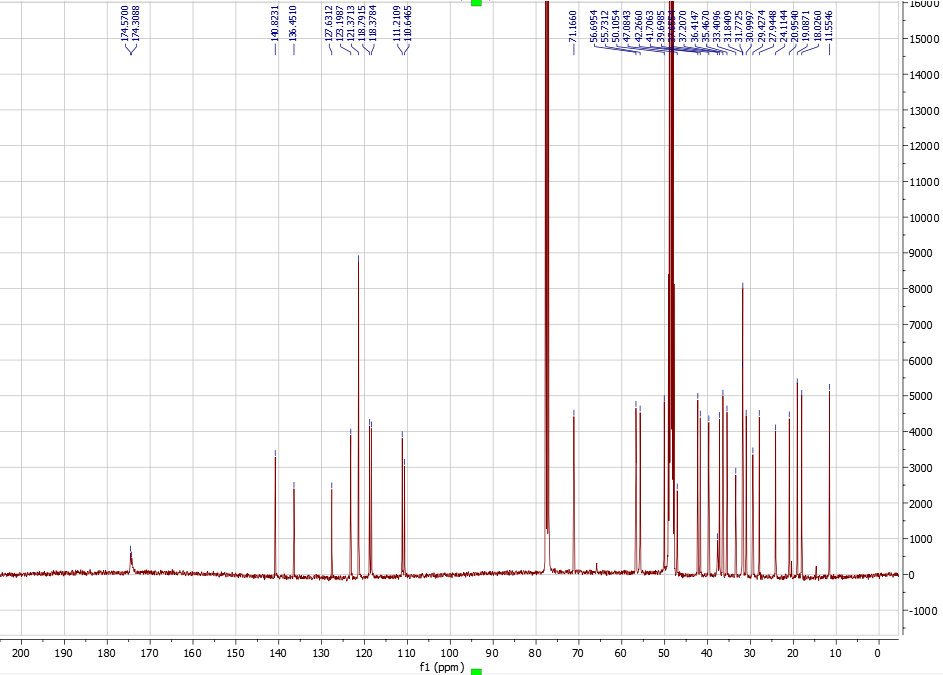


CDCl_3_

CD_3_OD

^13^C NMR (CD_3_OD/CDCl_3_,1/1 100 MHz) **UniPR!447**

Experimental high resolution mass values and isotopic distribution in ESI(+) for **UniPR1447** (upper) if compared to calculated value (lower spectrum).

**UniPR1449**

***N*-(3β-hydroxy-Δ^5^-cholen-24-oyl)-1-benzensulfonyl-L-β-homotryptophan (UniPR1449)**.

Compound **6** (100 mg, 0.14 mmol, 1 eq.) was dissolved in THF/H_2_O (1/1 v/v) and a solution of LiHMDS 1 M in THF (750 μL, 0.75 mmol, 5eq.) was added dropwise and stirred overnight at rt. Once the reaction is completed, the reaction was quenched and solution neutralized with AcOH and the solvent was removed under reduced pressure. The crude was dissolved in AcOEt and washed with acid water, Brine and Na_2_SO_4_. The final compound **UniPR1449** (75 mg, 0.1mmol, yield = 78%) was obtained after column chromatography DCM:MeOH:AcOH = 95:5:0.5 v/v/v. M.p = 136-139 °C. ^1^H NMR (CDCl_3_, 400 MHz,) δ 7.95 (d, *J* = 8.2 Hz, 1H), 7.87 – 7.78 (m, 2H), 7.63 (d, *J* = 7.7 Hz, 1H), 7.50 (t, *J* = 7.5 Hz, 1H), 7.45 – 7.36 (m, 3H), 7.30 (t, *J* = 7.7 Hz, 1H), 7.23 (m, 1H), 5.33 (d, *J* = 5.1 Hz, 1H), 4.51 (s, 1H), 3.58 – 3.48 (m, 1H), 3.10 – 2.81 (m, 2H), 2.54 – 2.35 (m, 2H), 2.33 – 2.15 (m, 3H), 2.02 – 1.86 (m, 6H), 1.85 – 1.67 (m, 3H), 1.60 – 1.33 (m, 8H), 1.31 – 1.0 (m, 8H), 0.98 (s, 3H), 0.88 (d, *J* = 6.5 Hz, 3H), 0.64 (s, 3H). ^13^C {^1^H} NMR (CDCl_3_, 100 MHz) δ 173.9, 171.1, 140.8, 137.9, 135.2, 133.8, 130.8, 129.3, 126.7, 125.0, 124.2, 123.5, 121.6, 119.8, 119.2, 113.7, 71.5, 56.7, 55.7, 50.1, 48.8, 45.6, 42.3, 42.0, 39.7, 37.2, 36.5, 35.5, 34.7, 33.6, 31.9, 31.8, 31.6, 31.4, 29.7, 29.2, 28.1, 24.2, 21.0, 19.3, 18.3, 11.8. ESI(+)-MS calc. for [C_42_H_54_N_2_O_6_+H]^+^: 715.3775 ; found: 715.3771. Error: -0.56 ppm. [α]_D_^25^ = -38.9° (c = 0.034 g/100 ml, MeOH).


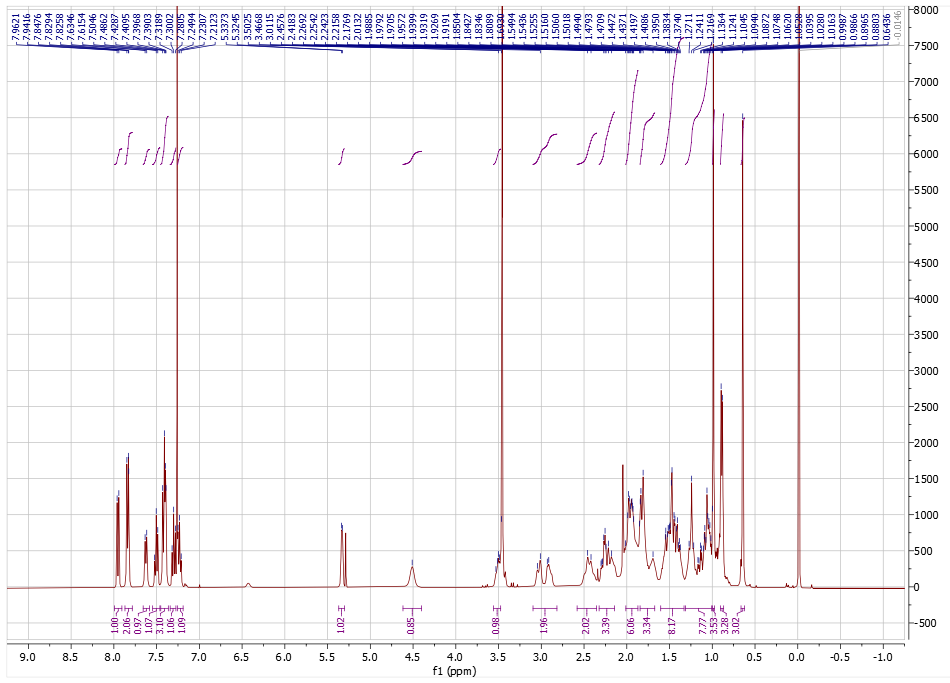


CDCl_3_

^1^H NMR (CDCl_3_, 400 MHz) **UniPR1449**


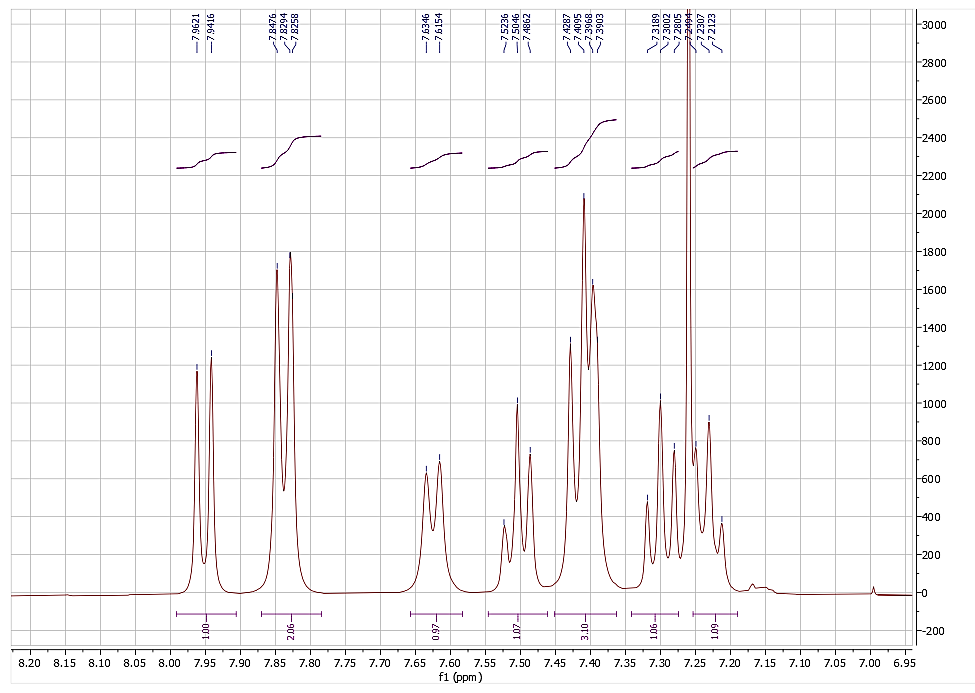


CDCl_3_

Magnification of the aromatic portion (8.20 – 7.0 ppm) of ^1^H NMR spectrum of **UniPR1449** (400 MHz, CDCl_3_)

CDCl_3_

^13^C NMR (CDCl_3_, 100 MHz) **UniPR1449**

Experimental high resolution mass values and isotopic distribution in ESI(+) for UniPR1449 (upper) if compared to calculated value (lower spectrum).

**Reaction Intermediates**

**N_β_-tert-butoxycarbonyl-L-β-homotryptophan methyl ester (4).** L-β-Homotryptophan hydrochloride (250 mg, 0.98 mmol, 1 eq.) was suspended in MeOH and stirred in an ice-bath. To this suspension, it was added SOCl_2_ (150 μL, 1.96 mmol, 2 eq.). Once obtained a solution, the reaction was set to reflux. The reaction was monitored with TLC and when completed it was quenched with a sat. solution of NaHCO_3_ and adjusted to basic pH. The solvent was removed under reduce pressure to obtain the methyl ester intermediate (yield = quant.). The crude was dissolved in dioxane and H_2_O, NaHCO3 (250 mg, 2.94 mmol, 3eq.) was added, followed by the addition of Boc_2_O (425 mg, 1.96 mmol, 2eq.) and stirred at room temperature. Upon completion of the reaction the solvent was removed under pressure, the crude was extracted with AcOEt and H_2_O, washed with brine and the organic phase treated with Na_2_SO_4_. The desired compound **4** (260 mg, yield = 80%) was obtained after purification with silica gel column chromatography (Hex/AcOEt 7/3). ^1^H NMR (CDCl_3_, 600 MHz) δ 8.18 (s, 1H), 7.64 (d, *J* = 7.9 Hz, 1H), 7.34 (d, *J* = 8.1 Hz, 1H), 7.19 (td, *J* = 7.6, 1.0 Hz, 1H), 7.12 (td, *J* = 7.6, 0.9 Hz, 1H), 7.02 (s, 1H), 5.14 – 5.00 (m, 1H), 4.31 (m, 1H), 3.66 (s, 3H), 3.14 – 2.93 (m, 2H), 2.51 (m, 2H), 1.43 (s, 9H). ^13^C {^1^H} NMR (CDCl_3_, 150 MHz) δ 172.40, 155.41, 136.34, 127.81, 122.92, 122.18, 119.63, 119.08, 111.85, 111.20, 79.40, 51.69, 48.27, 37.99, 30.00, 28.46.


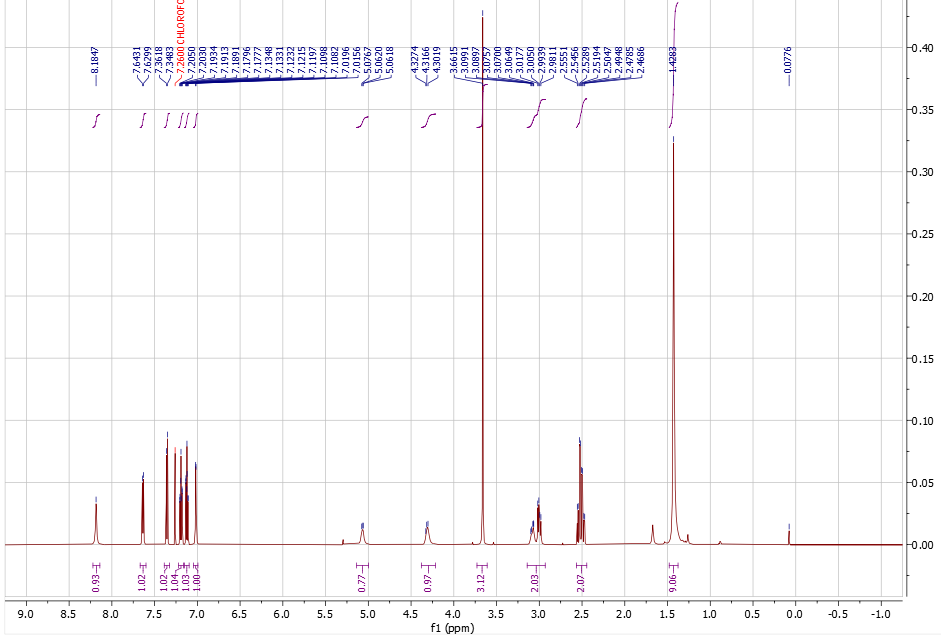


CDCl_3_

^1^H NMR (CDCl_3_, 600 MHz) **Compound 4**


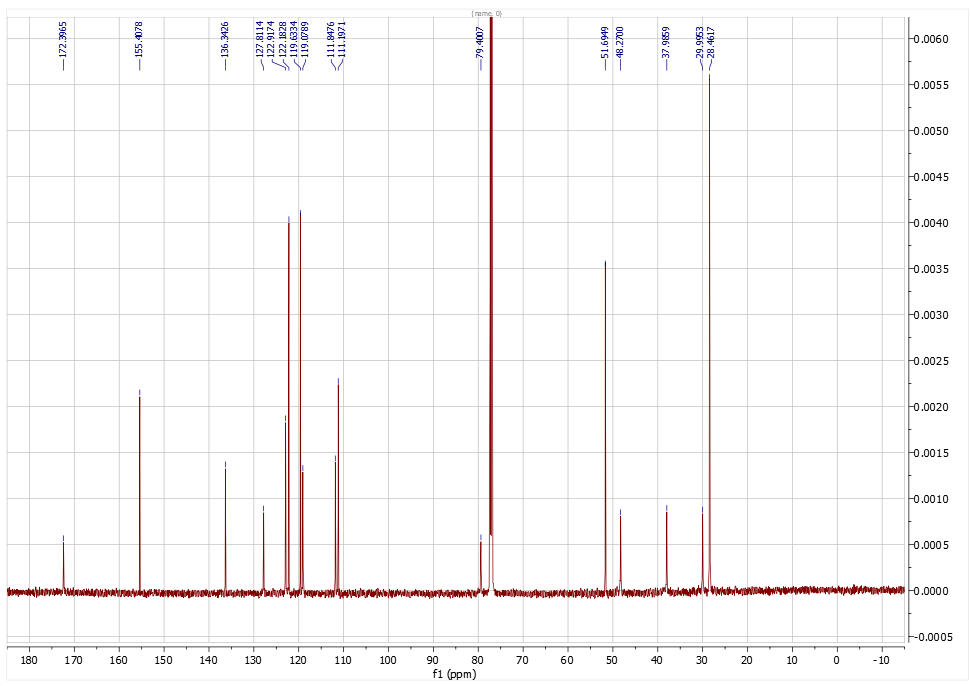


CDCl_3_

^13^C NMR (CDCl_3_, 150 MHz) **Compound 4**

**N_β_-*tert*-butoxycarbonyl-1-benzensulfonyl-L-β-homotryptophan methyl ester (5).** Compound **4** (250 mg, 0.75 mmol, 1 eq.) is dissolved in DCM (5 ml) and H_2_O (5 ml) is added. NaOH (36 mg, 0.9 mmol, 1.2 eq) and NBu_4_Cl (250 mg, 0.9 mmol, 1.2 eq.) are added to the biphasic mixture. After 15 min, a solution of PhSO_2_Cl (200 mg, 1.12 mmol, 1.5 eq) in DCM (2 ml) is added and the resulting mixture is stirred at room temperature. When the reaction is judged complete by TLC analysis, the phases are separated, the organic layer is collected and washed with brine and dried over NaSO_4_. After removal of the solvent under reduced pressure, the residue is purified by silica gel column chromatography (Toluene/AcOEt = 9/1) to furnish **5** (163 mg, yield =46%) as white foam. ^1^H NMR (CDCl_3_, 600 MHz) δ 7.97 (d, *J* = 8.3 Hz, 1H), 7.87 – 7.83 (m, 2H), 7.58 (d, *J* = 7.8 Hz, 1H), 7.53 – 7.50 (tt, *J*= 7.5, 1.2 Hz, 1H), 7.45 – 7.40 (m, 2H), 7.39 (s, 1H), 7.34 – 7.29 (td, *J* = 7.7, 1.0 Hz, 1H), 7.26 – 7.22 (td, *J* = 7.6, 1.0 Hz, 1H), 5.11 (d, *J* = 8.3 Hz, 1H), 4.27– 4.18 (m, 1H), 3.69 (s, 3H), 3.09 – 2.84 (m, 2H), 2.56 – 2.37 (m, 2H), 1.42 (s, 9H). ^13^C {^1^H} NMR (CDCl_3_, 150 MHz,) δ 172.08, 155.24, 138.24, 135.37, 133.81, 130.96, 129.31, 126.81, 125.02, 124.33, 123.48, 119.88, 119.19, 113.81, 79.66, 51.83, 47.31, 37.53, 29.77, 28.4.


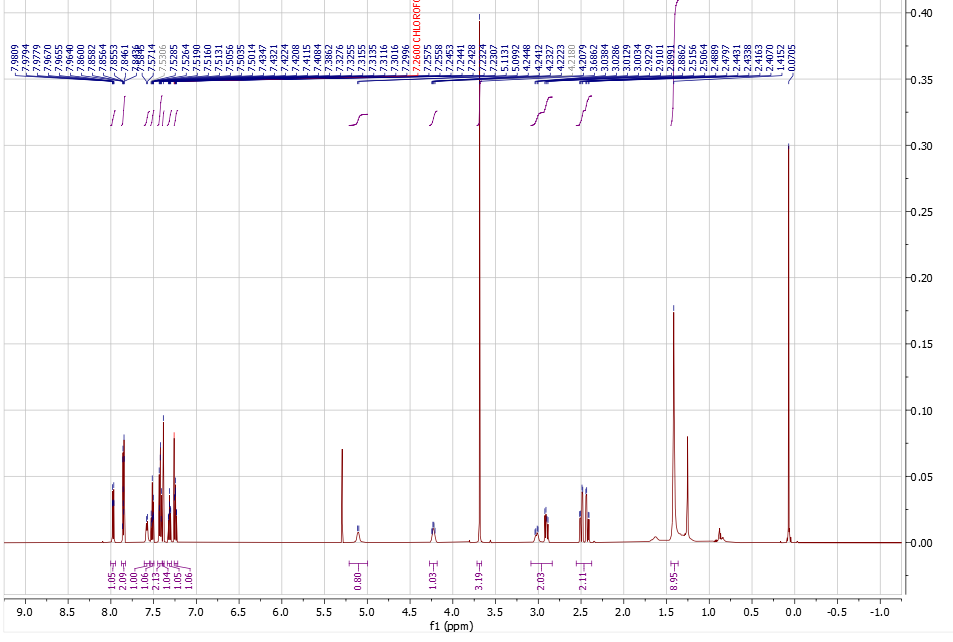


CDCl_3_

^1^H NMR (CDCl_3_, 600 MHz) **Compound 5**

^^
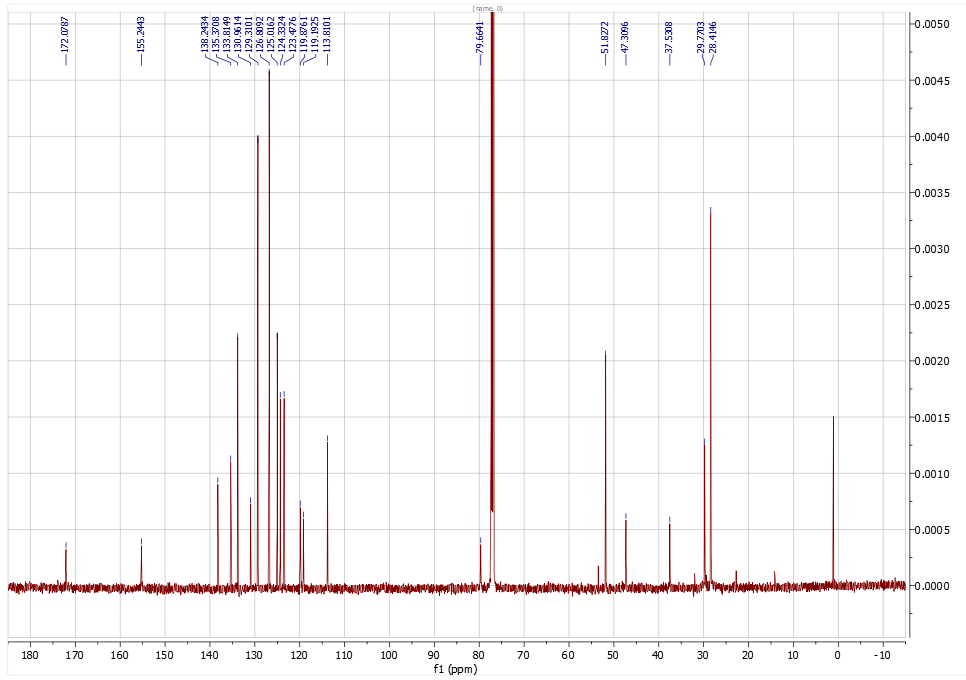


CDCl_3_

^13^C NMR (CDCl_3_, 150 MHz) **Compound 5**

***N*-(3β-hydroxy-Δ^5^-cholen-24-oyl)-1-benzensulfonyl-L-β-homotryptophan methyl ester (6).**

Compound **5** (150 mg, 0.31 mmol, 1eq.) was dissolved in 1 mL of DCM and chilled to 0° C. Trifluoroacetic acid (TFA, 1 mL) is added, and after 1h the reaction was quenched with saturated aqueous NaHCO_3_. The organic phase is washed with brine and evaporated. In a separate flask, cholenic acid (140 mg, 0.38 mmol, 1.2 eq.) in DMF (10.0 mL) at 0 °C is aaded with TBTU (*O*-(Benzotriazol-1-yl)-*N*,*N*,*N′*,*N′*,-tetramethyluronium tetrafluoroborate, 120 mg, 0.38 mmol, 1.2 eq.) and DIPEA (*N*,*N*-diisopropyl-*N*-ethylamine, 275 μL, 1.58 mmol, 5eq.). When a perfectly clear solution is obtained, the L-homotryptophan-N-indole-sulfonylated derivative obtained (92 mg, 0.24 mmol, 1 eq.) is added and the mixture is stirred for 16 hours. The mixture is poured over AcOEt (50 mL) and neutralized. The aqueous layer is discarded, and the organic layer is washed at first with brine. The organic phase is dried over Na_2_SO_4_ and evaporated under reduced pressure. The crude material thus obtained is purified by silica gel column chromatography eluting at DCM/AcOEt 8/2 (117 mg, yield = 52% over two steps). ^1^H NMR (CDCl_3_, 400 MHz) δ 7.96 (d, *J* = 8.3 Hz, 1H), 7.86 – 7.80 (m, 2H), 7.61 (d, *J* = 7.8 Hz, 1H), 7.54 – 7.47 (tt, *J* = 7.4, 1.2 Hz, 1H), 7.44 – 7.37 (m, 2H), 7.36 (s, 1H), 7.30 (td, *J* = 7.8, 1.2 Hz, 1H), 7.23 (td, *J* = 7.7, 1.1 Hz, 1H), 6.29 (d, *J* = 8.5 Hz, 1H), 5.37 – 5.28 (m, 1H), 4.57 – 4.46 (m, 1H), 3.68 (s, 3H), 3.57 – 3.42 (m, 1H), 3.10 – 2.78 (m, 2H), 2.52 – 2.37 (m, 2H), 2.31 – 2.11 (m, 4H), 2.05 – 1.89 (m, 3H), 1.85 – 1.77 (m, 3H), 1.76 – 1.65 (m, 1H), 1.60 – 1.34 (m, 8H), 1.31 – 1.24 (m, 2H), 1.18 – 1.00 (m, 5H), 0.98 (s, 3H), 0.89 (d, *J* = 6.5 Hz, 3H), 0.64 (s, 3H). ^13^C {^1^H} NMR (CDCl_3_, 100 MHz) δ 173.26, 172.34, 140.81, 138.09, 135.25, 133.78, 130.80, 129.25, 126.71, 125.02, 124.15, 123.48, 121.60, 119.86, 119.04, 113.71, 71.70, 56.71, 55.74, 51.84, 50.08, 45.68, 42.36, 42.27, 39.75, 37.27, 36.63, 36.49, 35.44, 33.62, 31.87, 31.62, 29.32, 28.14, 24.25, 21.06, 19.40, 18.39, 11.87.


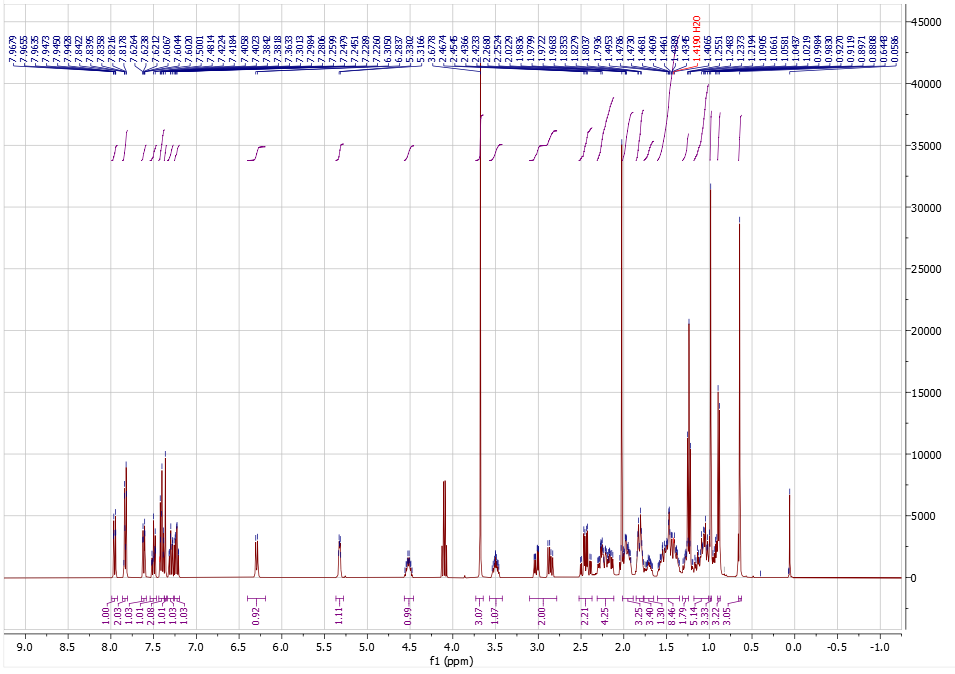


CDCl_3_

^1^H NMR (CDCl_3_, 400 MHz) **Compound 6**


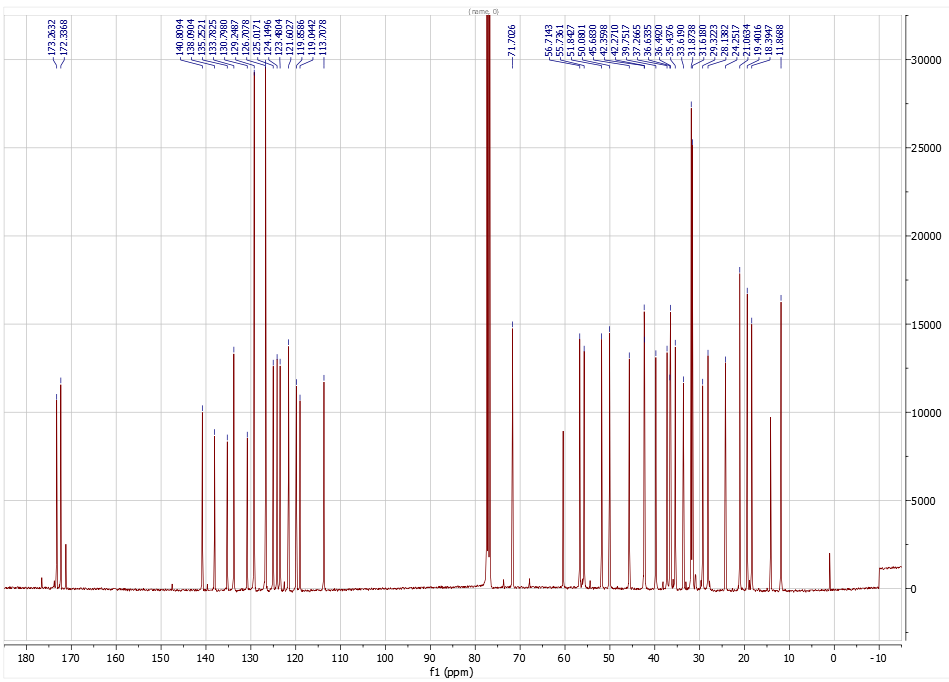


CDCl_3_

^13^C NMR (CDCl_3_, 100 MHz) **Compound 6**

#

# Full Scan HPLC/MS trace for UniPR1447

Full scan HPLC/MS trace in ESI(+) for UniPR1447 (lower trace) if compared to blank (upper trace).

| **RT (min)** | **Area** | **%Area** |
| --- | --- | --- |
| 8.41 | 363228538 | >99 |
| **SUM** | **363228538** | **>99** |

# Full Scan HPLC/MS trace for UniPR1449

Full scan HPLC/MS trace in ESI(+) for UniPR1449 (lower trace) if compared to blank (upper trace).

| **RT (min)** | **Area** | **%Area** |
| --- | --- | --- |
| 9.46 | 24189827 | 3.7 |
| 10.86 | 638299993 | 96.3 |
| **SUM** | **662489820** | **100** |
